# Curcuminoids as Modulators of EMT in Invasive Cancers: A Review of Molecular Targets With the Contribution of Malignant Mesothelioma Studies

**DOI:** 10.3389/fphar.2022.934534

**Published:** 2022-07-08

**Authors:** Daniel L. Pouliquen, Alice Boissard, Cécile Henry, Olivier Coqueret, Catherine Guette

**Affiliations:** ^1^ Inserm, CNRS, Nantes Université, CRCI2NA, Université d’Angers, Angers, France; ^2^ ICO, Inserm, CNRS, Nantes Université, CRCI2NA, Université d’Angers, Angers, France

**Keywords:** EMT cancer, curcuminoids cancer, cancer invasiveness, signaling pathway, tumor micro environment, molecular targets, malignant mesothelioma, biomarkers

## Abstract

Curcuminoids, which include natural acyclic diarylheptanoids and the synthetic analogs of curcumin, have considerable potential for fighting against all the characteristics of invasive cancers. The epithelial-to-mesenchymal transition (EMT) is a fundamental process for embryonic morphogenesis, however, the last decade has confirmed it orchestrates many features of cancer invasiveness, such as tumor cell stemness, metabolic rewiring, and drug resistance. A wealth of studies has revealed EMT in cancer is in fact driven by an increasing number of parameters, and thus understanding its complexity has now become a cornerstone for defining future therapeutic strategies dealing with cancer progression and metastasis. A specificity of curcuminoids is their ability to target multiple molecular targets, modulate several signaling pathways, modify tumor microenvironments and enhance the host’s immune response. Although the effects of curcumin on these various parameters have been the subject of many reviews, the role of curcuminoids against EMT in the context of cancer have never been reviewed so far. This review first provides an updated overview of all EMT drivers, including signaling pathways, transcription factors, non-coding RNAs (ncRNAs) and tumor microenvironment components, with a special focus on the most recent findings. Secondly, for each of these drivers the effects of curcumin/curcuminoids on specific molecular targets are analyzed. Finally, we address some common findings observed between data reported in the literature and the results of investigations we conducted on experimental malignant mesothelioma, a model of invasive cancer representing a useful tool for studies on EMT and cancer.

## 1 Introduction

Curcuminoids are plant secondary metabolites with a seven carbon skeleton possessing two phenyl rings at the 1- and 7-positions (acyclic diarylheptanoids), mainly isolated from the roots of plants of the *Curcuma* genus (Zingiberaceae) (Jahng and Park 2018). By extension, the term is now also used for synthetic analogs derived from the chemical structure of the most famous of these natural molecules for its applications in health, curcumin (Laali et al., 2019; Mishra et al., 2019). During the last decade, the inter-relationship between cancer stem cells (CSCs) and EMT, two potential targets of both synthetic and natural curcuminoids, has received special attention, and was the subject of several important reviews (Bao et al., 2012; Li and Zhang, 2014; McCarty 2012; Prud’homme 2012; Suresh et al., 2016).

Most initial studies were not specifically focused on the effects of curcuminoids, but rather on those of a set of natural compounds, mostly polyphenols, that included curcumin. Benefits in chemoprevention were particularly documented (Ahmad et al., 2012; Kallifatidis et al., 2016), and the involvement of microRNAs (miRNAs) in CSCs and EMT regulation was especially reviewed, which represented another target for curcumin (Wang et al., 2010; Sethi et al., 2013; Suresh et al., 2016). Among self-renewal pathways, the impact of curcumin on Hedgehog signaling was also investigated (Sarkar et al., 2010; Li et al., 2012; Sun et al., 2013). Progressively, the potential of curcumin to reverse EMT was confirmed (Avila-Carrasco et al., 2019; Bahrami et al., 2019), while the field of potential applications in cancer, combined with other therapeutic tools, moved from chemoprevention to therapy (Varghese et al., 2018; Shen Y. W. et al., 2021; Ashrafizadeh et al., 2020). Moreover, the modulation of cancer aggressiveness and targeting of CSCs produced by curcumin when associated with other phytochemicals (defined as biologically active compounds found in plants) has been established (Mitra and Bhattacharya, 2020; Naujokat and McKee, 2021). In parallel, the involvement of tumor related ncRNAs was also investigated in the multiple effects of curcuminoids on the regulation of cancer progression (Wang H. et al., 2021a). Finally, among other natural compounds, the essential signaling pathways associated with EMT on which curcumin produces its effects was reviewed in the peculiar context of breast cancer (Lu Y. et al., 2021).

Here, we first present an overview of recent breakthroughs in our understanding of the complex regulation of EMT in cancer. Next, for each signaling pathway and the tumor microenvironment components associated with this process, the detailed effects of curcumin/curcuminoids reported in the literature were reviewed. Finally, we address some common findings between data reported in the literature and the results of investigations conducted on experimental malignant mesothelioma (MM), a model of invasive cancer that is a useful tool for studies on EMT and cancer.

## 2 Materials and Methods

**FIGURE 1:**
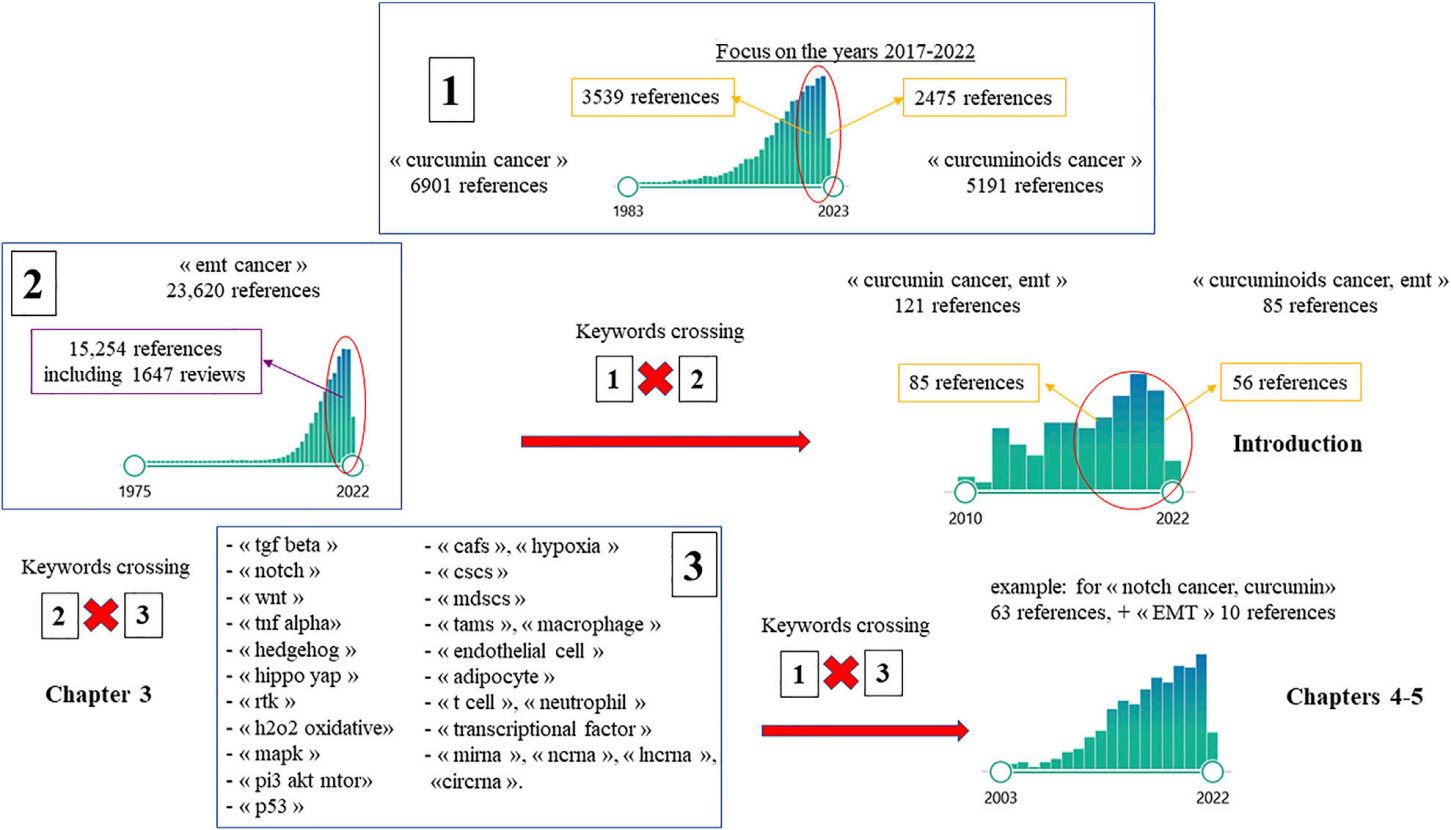
Flow diagram of the study selection process (detailed in the text, chapter 2).

**FIGURE 2:**
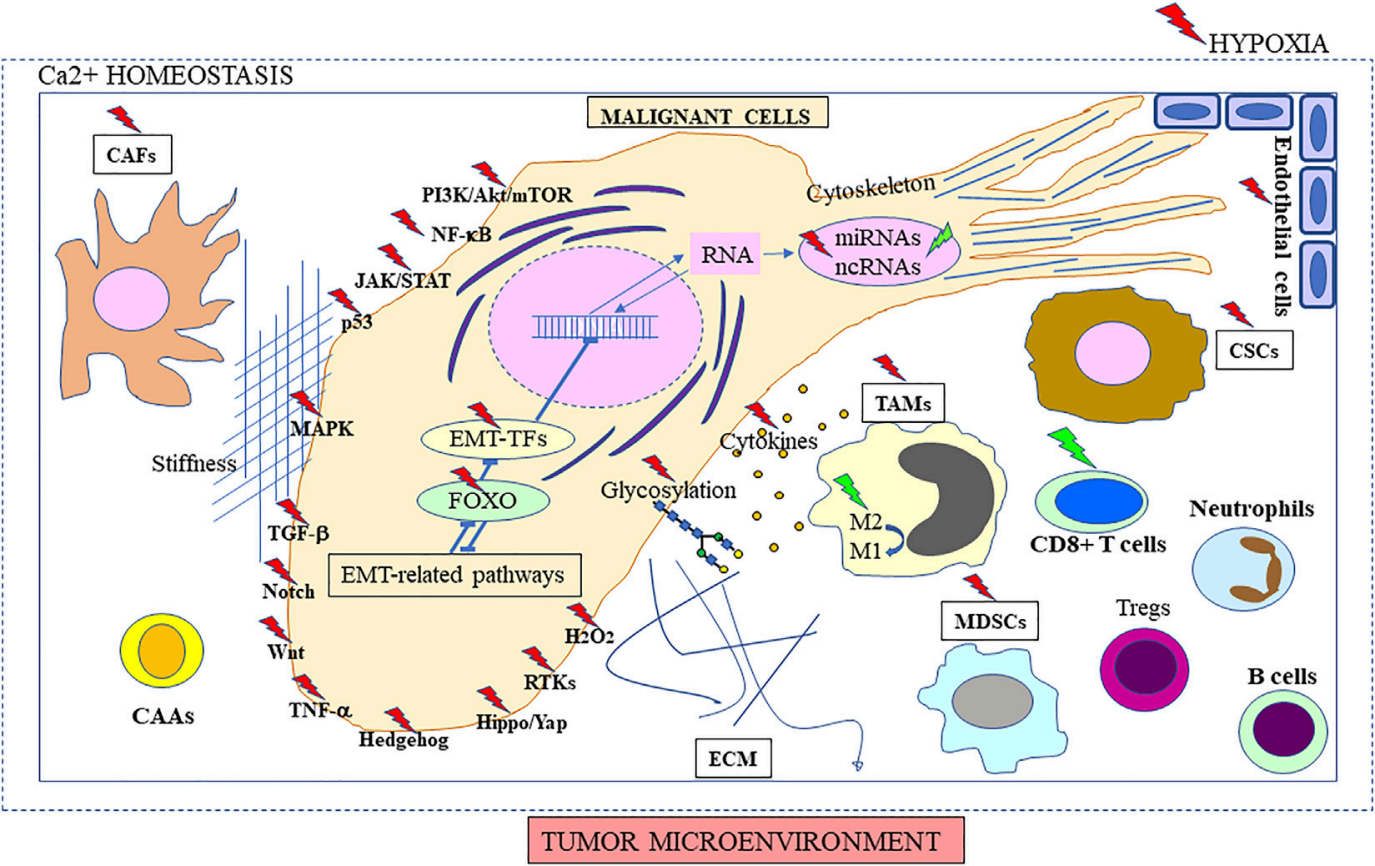
Diversity of EMT drivers in cancer. Targets of curcumin/curcuminoid reported in the literature are indicated with green (positive regulation) or red thunderstorms (negative regulation), including molecular components of signaling pathways, and gene expression/regulation of stromal and malignant cells, and secreted proteins. The role of the different epithelial-to-mesenchymal transition (EMT) drivers is detailed in chapter 3, and molecular targets are described in chapters 4-6 (illustrated in Figures 3, 4). Factors such as hypoxia and its interplay with tumor calcium homeostasis may influence intercellular crosstalk leading to extracellular matrix (ECM) remodeling, and increased migratory and metastatic behavior, representing an additional physiological target (developed in Sections 3.2, 3.5, 3.6, 3.10 and chapters 4 and 6). The role of stiffness is also explained in the fourth paragraph of Section 3.10 and last paragraph of chapter 6. M2 → M1 symbolizes imbalance between the two macrophage subtypes producing anti-inflammatory and pro-inflammatory cytokines, respectively.

The study design was based on a review of the current literature searched on PubMed database using different combinations of keywords, with a special focus on the period 2017-2022 (Figure 1). In order to provide an update of the main recent findings on EMT driving parameters, the keyword “EMT cancer” was crossed with those of the signaling pathways, tumor microenvironment components and other specific EMT drivers (Figure 2). All relevant articles published in English were considered, with a special focus on reviews or works providing important breakthroughs in the field (covered in chapter 3). In a second step the keywords “curcumin cancer” or “curcuminoids cancer” were crossed with “EMT cancer” and all recent reports highlighting important findings were included (chapters 4-5). Finally, these reference lists were crossed with the keyword “malignant mesothelioma” and additional information relevant to this peculiar cancer type was reviewed in chapter 6.

## 3 Overview of EMT Driving Parameters in Cancer, an Update

The EMT process and its involvement in certain pathological events, such as cancer cell metastasis, were first described in the pioneering work by Boyer and colleagues (Boyer and Thiery, 1993). EMT was then recognized as playing a fundamental role during the early stages of invasion and metastasis of carcinoma cells (Boyer et al., 2000). However, in the mid-2010s researchers questioned the real role of EMT in the metastatic process and the debate led to a clarification of many issues related to EMT in cancer biology (Brabletz et al., 2018). Subsequently, the complexity of EMT regulation and its coordination at multiple levels started to be deciphered (Simeone et al., 2019). Progressively, its potential to yield novel therapies prone to overwhelm therapeutic resistance and to improve the management of high-grade cancers was also revealed (Zhang and Weinberg, 2018). To date, EMT appears to orchestrate a wide variety of complementary cancer features, including tumorigenicity, stemness, resistance to therapy and adaptation to changes in the microenvironment (Brabletz et al., 2021). Moreover, beside its role in embryogenesis and wound healing, recent investigations on EMT and its reverse process, mesenchymal-epithelial transition (MET) in cancer have demonstrated their plasticity and the existence of a multilayer regulatory network allowing cells to exhibit multiple hybrid E/M states, rather than a binary switch between epithelial and mesenchymal states (Bornes et al., 2021).

To take just the specific case of breast cancer as an example, the regulation of EMT appears to be centered on a list of different signaling pathways that include transforming Growth Factor Beta (TGF-β), Notch, Wnt, tumor necrosis factor alpha (TNF-α), Hedgehog, and receptor tyrosine kinases (RTKs) (Buyuk et al., 2021) (Figure 2). In addition to Hippo/yap, nuclear factor-kappa B (NF-κB), janus kinases/signal transducer and activator of transcription proteins (JAK/STAT), mitogen-activated protein kinases (MAPK), and phosphatidylinositol 3-kinase (PI3K)/Akt/mechanistic target of rapamycin (mTOR), investigations on each of these pathways have been the subject of new developments in the last 3 years and are reviewed below. Meanwhile, the role of potential additional factors that drive EMT in cancer (Figure 2) was also highlighted, namely transcriptional factors (TFs) (Vishnoi et al., 2020), translation initiation mechanisms (Bera and Lewis, 2020), miRNAs (Pan et al., 2021) and circular RNAs (circRNAs) (Jiang M. et al., 2021), hypoxia (Peng P. H. et al., 2021), inflammation (Chattopadhyay et al., 2021), and stromal components like tumor-associated macrophages (TAMs) (Han et al., 2021) and cancer-associated fibroblasts (CAFs) (Asif et al., 2021). Other emerging or developing research fields were represented by cancer-associated adipocytes (Liu et al., 2020), endothelial cells and their transformation via endothelial-to-mesenchymal transition (EndMT) (Yoshimatsu and Watabe, 2022), and vascular mimicry (Sun et al., 2017). Additionally, the orchestration of multiple EMT-associated pathways and EMT-associated TFs by Forkhead box O (FOXO) proteins was also documented (Ma et al., 2018). The links with glycobiology (Pucci et al., 2021), Ca^2+^ homeostasis (Jones and Hazlehurst, 2021) and the cytoskeleton (Leggett et al., 2021) were also explored, while the complexity of its interrelationships with CSCs (Roy et al., 2021), p53 (Parfenyev et al., 2021) or H_2_O_2_ signaling (Milton and Konrad, 2022), and their consequences on the generation of circulating tumor cells (CTCs) (Topa et al., 2022) or occurrence of a collective invasion process (Nagai et al., 2020) were updated. Finally, as EMT is involved in such a broad range of processes associated with malignant transformation and invasiveness, it has become an increasingly interesting target for the development of new therapeutic strategies (Dudas et al., 2020; Jonckheere et al., 2022).

### 3.1 TGF-β

TGF-β was one of the first growth-active polypeptides fitting the definition of growth factors that was recognized as being associated with the neoplastic process, and its distinction with TGF-α was clearly established based on molecular composition, biological response produced and membrane receptor binding (Goustin et al., 1986). It was also one of the earliest factors described as being associated with the EMT process in cancer (Hay, 1995), a process which was shown to lead to drug resistance *in vivo*, drug sensitivity in the resistant tumors being restored using neutralizing antibodies directed against this growth factor (Teicher et al., 1996). These pioneering investigations led to the discovery of the function of decorin, a small cellular or pericellular matrix proteoglycan, as an inhibitor of TGF-β1 (Teicher et al., 1997). An overview of the role of this signaling pathway in tumor initiation and progression has previously been given by Liu et al. (2018), and recently updated by Stuelten and Zhang (Stuelten and Zhang, 2021).

**FIGURE 3:**
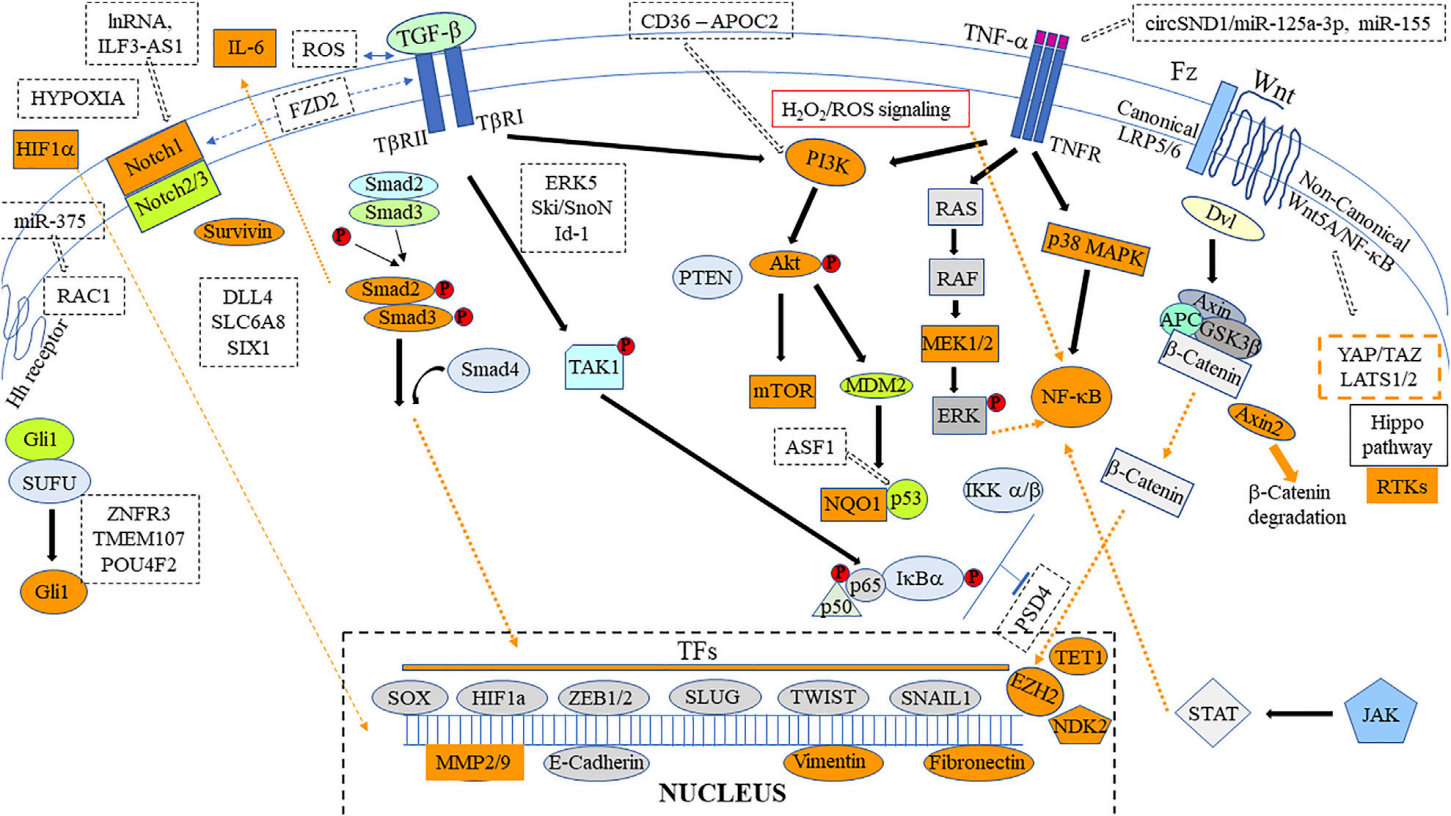
Main signaling pathways involved in curcumin/curcuminoid anticancer effects. Targets reported in the literature are identified as orange boxes/dashed lines. Some important new recent proteins/microRNAs (miRNAs) or non-coding RNAs (ncRNAs) identified to be involved in the regulation of peculiar signaling pathways are indicated in dashed black boxes. Other crucial potential molecular targets/signaling pathways detailed in Sections 3.5–3.9 and chapters 4-6 are omitted for clarity.

Recent findings on the involvement of this signaling pathway in EMT and linked with cancer invasiveness demonstrated crosstalk with invasive stress. It was found that TGF-β regulates the production of reactive oxygen species (ROS) or modulates antioxidant systems and redox-sensitive transcription factors in cancer cell metabolism by increasing the redox imbalance. Moreover, increased ROS production may directly induce TGF-β expression at a nuclear level (Ramundo et al., 2021). Kumari et al. (2021) also reported that oxidative stress and hypoxia lead to the accumulation of misfolded proteins in the endoplasmic reticulum (ER), thus inducing ER stress (ERS), which is modulated by G protein-coupled receptors (GPCRs) via ERS sensors, to support cancer cell survival and inhibit cell death. Additionally, these authors stated that TGF-β regulates EMT both at the transcriptional and post-transcriptional levels in ovarian cancer progression (Kumari et al., 2021b). Dash et al. (2021) also revealed the existence of a link between TNF-α and TGF-β under the governance of various downstream signals such as the member of the MAP3K kinase family TAK1 (Figure 3), also known as MAP3K7. Another kinase under investigation is extracellular signal-regulated kinase 5 (ERK5), the newest member of the MAPK family and an essential regulator of cancer progression, tumor relapse and poor patient survival, which can be activated both by TNF-α and TGF-β (Bhatt et al., 2021). Two other negative regulators of TGF-β signaling, Ski and Ski-related novel protein N (SnoN) were also reported, while TGF-β1 was suggested as being an upstream effector of Id-1, a protein belonging to the helix-loop-helix (HLH) transcription factor family, for its action in promoting cell survival and EMT in premalignant prostate epithelial progenitors (Figure 3). The dual role that TGF-β plays in prostate cancer, inhibiting cell proliferation in normal and early-stage cancer cells, but promoting cancer progression in later stages of the disease, was found to be related to an increase in the stability of the tumor suppressor gene phosphatase and TENsin homolog (PTEN) in the first case, while enhancing the effect of TGF-β on the deletion of PTEN observed in metastatic prostate cancer cells (Thompson-Elliott et al., 2021). Finally, selective inhibitors of the TGF-β signaling pathway are now under evaluation, showing early promise. However, the paradoxical role this growth factor may play in the tumor microenvironment, for example in the case of pancreatic adenocarcinoma, has led to questions regarding the potential biological consequences of inactivating selected components from this pathway, to identify the most effective combination therapy (Principe et al., 2021).

### 3.2 Notch

The involvement of the Notch signaling pathway in both embryogenesis and tumorigenesis has been known since 1951, however a growing interest in this topic started at the end of the 1990s. Subsequently, its link with EMT was first reviewed in 2004 (Grego-Bessa et al., 2004), emphasizing the role of cadherin downregulation. Notch was particularly associated with the maintenance of an uncommitted stem cell-like state in the beginning of the 2000s (Radtke and Raj, 2003). Among recent findings (Figure 3), a link with Hypoxia-inducible factor 1-alpha (HIF-1α) and estrogen signaling was established (De Francesco et al., 2018), hypoxia-associated EMT representing one of three modules driving the Notch-mediated EMT (Kar et al., 2019). Consequently, innovative treatment strategies targeting signaling pathways such as Notch have been proposed to control stem-cell replication, survival, and differentiation (Venkatesh et al., 2018). The complex connectivity of Notch with other signaling pathways, especially Wnt, have just started to be deciphered, highlighting its variability depending on the different contexts (Misiorek et al., 2021). Meanwhile, other authors focused on the interactions of Notch receptors with their ligands, trying to understand how their precise role could represent new targets, and to determine a patient’s response to therapies (Kalafut et al., 2021).

Among the crosstalk investigated between different signaling pathways, one member of the Frizzled receptor family (FZD), FZD2, was recently identified as mechanically promoting the TGF-β1-induced EMT process in breast cancer by activating Notch (Tuluhong et al., 2021). Additional breakthroughs have been revealed by bioinformatic analysis, showing that Notch 2/3 receptors and delta-like 4 Notch ligand (DLL4) differentiated non-cancerous samples from cancers, representing drivers of Notch signaling in bladder cancer (Zhang C. et al., 2021). Two other studies have demonstrated that SLC6A8 (Solute carrier family 6 member 8), a member of the γ-aminobutyric acid (GABA) transporter family (Feng et al., 2021) and Sine oculis homeobox homolog 1 (SIX1), a developmentally restricted transcriptional regulator (Huang et al., 2022), were involved in activation of Notch in non-small lung cancer. In the field of long non-coding RNAs (lnRNAs), ILF3-AS1, the antisense RNA of interleukin enhancer-binding factor 3 (ILF3), was reported to promote hepatocarcinoma progression via the Notch pathway (Yan et al., 2022) (Figure 3). Finally, another interesting finding was the observation of an EMT process activated via Notch signaling under conditions of intratumoral heterogeneity caused by chemotherapy, both in senescent and adjacent non-senescent breast cancer cells, which supported the promising prospects of inhibitors of this pathway in the treatment of breast cancer recurrence (Zhang N. et al., 2021).

### 3.3 Wnt

The interest in this signaling pathway in cancer emerged with the discovery in 1987 of a homology between the *int-1* mouse oncogene (activated in mouse mammary tumors) and a Drosophila gene called *wing-less* (Rijsewijk et al., 1987). Subsequently, Savagner (2001) suggested that the Wnt pathway could represent an essential step in the massive cytoskeleton reorganization observed *in vivo* during EMT. A pivotal function for β-catenin, a member of adherens junctions translocated to the nucleus, was then established in the EMT process, demonstrating that a critical threshold must be reached for the activation of certain target genes involved in cell motility during tumorigenesis (Müller et al., 2002). Given the complexity of this pathway, with about 19 genes encoding glycolipoproteins connected to various receptors that stimulate different intracellular pathways, these latter were divided into canonical (β-catenin dependent) and non-canonical (β-catenin independent) (Niehrs, 2012).

The first category is activated by the interaction of Wnt with a Frizzled receptor (Fz) and lipoprotein receptor-related proteins LRP5/LRP6, while the second category utilizes downstream signaling means classified according to the type of Wnt receptor and co-receptor they employ and the downstream receptor they pair with (Patel et al., 2019). Among the second category, the role of the Wnt5a/NF-κB has been particularly investigated in the context of studies on tumor microenvironment, revealing how an aberrant expression of Wnt5a is immunosuppressive (Lopez-Bergami and Barbero, 2020). In bone metastasis from prostate cancer, the Wnt receptor FZD4 was also reported to play an important role together with Wnt5a (Kaplan et al., 2021). Complementary investigations on this ligand suggest that Wnt5a is integrated into a larger regulatory circuit involving β-catenin, YAP/TAZ, and large tumor suppressor kinase 1/2 (LATS1/2) (Figure 3), with Wnt5a and yes-associated protein 1 (YAP1) both being regulated at the transcriptional level by both Wnt and Hippo pathways (Astudillo 2021). However, the extent of crosstalk between the Wnt pathway and the other multiple developmental signaling pathways are far from being completely understood as Merikhian et al. recently highlighted the complex interaction with the transactivated RTKs signaling pathway and lymphocytic infiltration in the context of EMT (Merikhian et al., 2021). Whatever type of cancer considered, activation of the Wnt pathway and EMT always presents deleterious consequences for patients, with the potential emergence of drug and/or immunoresistance in triple negative breast cancer (Merikhian et al., 2021), or hepatocellular carcinoma (Paskeh et al., 2021). The Wnt/β-catenin signaling pathway was shown to regulate stemness, for example in ovarian (Teeuwssen and Fodde, 2019), prostate (Kaplan et al., 2021) and colon cancer (Cheriyamundath and Ben-Ze’ev, 2021). Other biomarkers of the Wnt cascade, E-cadherin, vimentin, Adenomatous polyposis coli (APC), Snail and N-cadherin were revealed to be of importance in the carcinogenesis of other cancer types, such as oral squamous cell carcinoma (Bai et al., 2020). Finally, among additional biomarkers related to the Wnt pathway and EMT in invasive cancers, miRNAs and their upstream regulators circRNAs and lncRNAs appear to be crucial (Lei et al., 2020; Shao et al., 2020).

### 3.4 TNF-α

Several successive investigations conducted in 1980 on BCG-infected mice showed the capacity to induce the release into the serum of a substance named tumor necrosis factor, TNF, which mimicked the tumor-necrotizing action of endotoxin and was inhibitory or cytotoxic to a range of tumor cell lines (Oettgen et al., 1980). About 3 decades later, Balkwill reviewed the long history of the discovery of this cytokine, which acted primarily though TNFR1 (TNF receptor 1) following autocrine and paracrine production with crosstalk between malignant cells, myeloid and other cells from the tumor environment. At this stage, EMT was already mentioned in the list of changes produced by TNF in malignant cells linked with increased aggressiveness (Balkwill, 2009). The role of TNF in EMT and the direct effect of monocytes on the motility of pancreatic cancer cells was then investigated, revealing a phenotypic transition that was attenuated when cancer cells and monocytes were co-cultured in the presence of inhibitors of TNF production and anti-TNF antibodies (Baran et al., 2009).

The next decade was characterized by considerable interest in crosstalk routes between malignant cells and mesenchymal stromal cells (MSCs), some reports focusing on the specific role of TNF-α. In trying to improve their phenotyping and understanding of functional differences according to their tissue of origin, studies on MSCs concluded there was strong evidence that their functional heterogeneity was influenced by long-term exposure to a cancerous EMT environment, leading in some cases to their dedifferentiation into carcinoma-associated fibroblasts, changing immunomodulatory properties, cytokine profiles, and even increasing radio-/chemotherapy resistance (Böhrnsen et al., 2020). In the case of gastric cancer, Zhou Q. et al. (2020) highlighted the fact that among tumor-stroma interactions, the upregulation of interleukin 33 (IL-33) and its receptor suppressor of tumorigenicity 2 (ST2L) led to the production of Il-33 derived CAFS, which enhanced the migration and invasion of cancer cells by inducing EMT through activation of the ERK1/2-specific protein 1 (SP1)-zinc finger E-box binding homeobox 2 (ZEB2) pathway in a ST2L-dependent manner. Another report demonstrated that TNF-α activated two parallel signal transduction pathways, PI3K/Akt and p38 MAPK, then stimulating downstream NF-κB pathway p65 into the nucleus to activate C-X-C motif chemokine ligand (CXCL10) transcription in colon cancer cells (Wang Z. et al., 2021a). Moreover, in prostate cancer, recent studies on the activation of the NF-κB p65 signaling pathway by TNF-α identified the upregulation of the phosphorylated forms of two proteins, Ikappa B kinase (p-IKK) and inhibitor of nuclear factor kappa B (p-IκBα), (Wang M. et al., 2020). Additionally, Shi et al. (2020) found that the NF-κB p65 signaling pathway, when activated, epigenetically repressed pleckstrin and Sec7 domain-containing 4 (PSD4), a tumor suppressor gene that inhibits the proliferation, migration, and invasiveness of hepatocellular carcinoma (Figure 3). Finally, to study mechanisms of TNF-α induced EMT in thyroid cancer cells, Lv et al. (2021) demonstrated that only NF-κB inhibitors reversed the expression of the transcription factors.

In parallel, as circular RNAs appeared to play an important role in the relationship between inflammation and cancer, TNF-α was found to induce the expression of circSND1 through the transcription factor NF-κB, its function being exerted via a TNF-α/NF-κB/circSND1/miR-125a-3p/FUT6/NF-κB positive regulatory circuit (Bai et al., 2021). TNF-α also induces the upregulation of miR-155 in osteosarcoma cells, producing another feedback regulatory loop that promotes the acquisition of a CSC phenotype (Yao et al., 2020) (Figure 3). Evaluating the inhibitory effects of natural drugs on the activation of NF-κB produced by TNF-α led to identifying cyclooxygenase 2 (COX-2), C-myc, MMP-2, MMP-9 (metalloproteinases), intercellular adhesion molecule (ICAM-1) and vascular endothelial cell growth factor (VEGF) as representative downstream molecules involved in cancer invasion associated with EMT (Paramanantham et al., 2020). TNF-α induced EMT also appeared to upregulate immune modulators, including programmed death-ligands PD-L1, PD-L2, cluster of differentiation (CD) 73 and B7 Homolog 3 (B7-H3) (Shrestha et al., 2020). Moreover, Takahashi et al. (2020) revealed how TNF-α is involved in TGF-β-induced EndMT in endothelial cells, leading to the formation of CAFs.

### 3.5 Hedgehog

Early findings were related to a form of skin cancer called Gorlin’s syndrome, first detailed in the 1960s, with patients affected by a variety of additional malformations including craniofacial and brain abnormalities, suggesting the related gene would play a role in embryonic development (Currie, 1998). Given that Hedgehog signaling had a major impact on EMT in cancer, its activity being initially associated with metastatic ability in prostate carcinoma cell lines (Karhadkar et al., 2004), it corresponded to an essential pathway for stem cell function, together with Wnt and Notch (Huber et al., 2005). Consequently, this pathway represented an interesting target for overcoming treatment resistance for many invasive cancers (Gan and Jimeno, 2016). Its role in bladder (Syed et al., 2016) and triple negative breast cancer stemness and tumorigenesis (Habid and O’Shaughnessy, 2016), was reviewed, and the prognostic value of glioma-associated oncogene (Gli1), an essential mediator of this pathway, was emphasized (Rokkam et al., 2020). In lung cancer, the Hedgehog pathway mediated epidermal growth factor receptor tyrosine kinase inhibitor resistance, in association with EMT, however Gli1 knockdown reversed this resistance (Chen et al., 2022). In MM cells, targeting this signaling component, Gli1 or its target gene *Myc* inhibited cell viability and spheroid formation (Sementino et al., 2022). The activation of the Hedgehog/Gli1 signal pathway is suppressed by elevating the expression of the transmembrane E3 ubiquitin ligase (ZNRF3), which mediates Wnt proteins. Interestingly, Wang Z. et al. (2021b) demonstrated that RSPO2, a member of the R-spondin-2 protein family which plays an essential role in stem cell survival, when silenced inhibits the tumorigenicity of nasopharyngeal carcinoma via the zinc and ring finger 3 (ZNRF3)/Hedgehog-Gli1 axis. Another player, transmembrane protein 107 (TMEM107), whose function was previously unknown, was found to inhibit EMT and invasion in lung cancer through regulating the Hedgehog pathway (Xu H. et al., 2021). Among TFs, one member of the POU homeodomain family, POU4F2, also appeared to positively regulate Hedgehog (Guo et al., 2021). The link with hypoxia was also documented with the study of the effects of a natural lignan, saurochinone, which abrogated hypoxia-induced invasion and EMT in osteosarcoma cells via inactivation of this pathway (Zhou and He, 2021). Protein-protein interaction analysis allowed to identify another transcription factor, Gli2, which appeared to be regulated by miR-636 (Ma et al., 2021). In this field of research, miRNA-150 was found to repress both Wnt and Hedgehog pathways in gastric cancer (Peng Y. et al., 2021), while miR-375 regulated the Hedgehog pathway *via* Ras-related C3 botulinum toxin substrate 1 (RAC1) (Liang et al., 2021) (Figure 3). Finally, among lncRNAs, the intergenic LINC01426 was reported to promote the progression and stemness of lung cancer, an interaction with a member of the histone deubiquitinating complex Spt-Ada-Gcn5 acetyltransferase (SAGA), ubiquitin specific peptidase 22 (USP22), being suggested (Liu X. et al., 2021).

### 3.6 Hippo/yap and RTKs

The Hippo signaling pathway, which controls organ size, involves a kinase cascade involving three protein kinases and many genes recognized as tumor suppressors. In one of the pioneering studies, Overholtzer et al. (2006) reported how the wild-type gene *YAP*, which regulated proliferation and apoptosis in *Drosophila*, had potent oncogenic potential and induced phenotypic alterations including EMT. Mechanically, the epidermal growth factor receptor (EGFR) ligand amphiregulin (AREG), which appeared to be a target for the transcriptional co-activator YAP, was identified as a downstream effector of the Hippo pathway (Zhang et al., 2009). The crucial role of the interaction between YAP and the nuclear transcriptional enhancer factors (TEADs), leading to the induction of invasive pseudopodia in tumor cells, was recently reviewed (Li et al., 2021). In fact, the transcriptional activity of YAP appears to be controlled by a growing number of upstream regulators including several members of the WWC-and-C2 domain-containing protein family (Höffken et al., 2021). Recent findings have highlighted the role of several modulators of the Hippo signaling pathway, including the high mobility group AT-hook 2 (HMGA2), a DNA binding protein (Xu J. et al., 2021), the scribble planar cell polarity protein (SCRIB) (Shen H. et al., 2021), and GABABR1, one of the two subunits making up the GABA_B_ receptor (Wang H. et al., 2021b). The upregulation of receptor of collagen I, Discoidin Domain Receptor Tyrosine Kinase 2 (DDR2), also confers a susceptibility to ferroptosis via the Hippo pathway (Lin C. C. et al., 2021). In the field of RNAs, miR-223-3p (Du et al., 2021) and long non-coding LINC00649 (Wang H. et al., 2021c) were identified as regulators. Jiang L. et al. (2021) revealed that YAP upregulation induces proliferation, migration and EMT progression of colorectal cancer cells through the Glut3/AMPK signaling pathway. Finally, the links with other pathways were first established by demonstrating that hypoxia induced the upregulation of the actin-bundling protein Fascin-1 (regulated by the Akt/Rac1 signaling), thereby mediating both actin cytoskeleton rearrangement and Hippo activation (Pu et al., 2021). Secondly, another link with the Wnt pathway was established with the work of Yang et al. (2021), who reported that a member of the potassium channel tetramerization domain family, KCTD11, inhibited β-catenin nuclear translocation and Wnt pathway activity, which further inhibited the Hippo pathway.

The Hippo signaling pathway is mainly carried out by two activated pathways involving RTKs, the RTK/RAS/PI3K and the RTK-RAS-MAPK pathways (Azad et al., 2020). RTKs also affect cadherin cell-cell adhesion (Kaszak et al., 2020). At the beginning of the last decade, Thomson et al. (2011) reported a loss of autocrine RTK signaling networks, within the complex EMT associated cell-cell junction and cell-adhesion/extracellular matrix (ECM) changes, grouped by system and assembled by protein-protein contacts, through EGFR/ErbB2/ErbB3, Met/Ron and insulin-like growth factor 1 receptor (IGF1R) signaling networks. Subsequently, Antony et al., in reviewing the signaling schemas enabling EMT, pointed to the amplified signaling of the Tyrosine-protein kinase receptor UFO (encoded by the *AXL* gene), and the extensive crosstalk observed with other RTKs such as EGFR, cMET and HER2, in the subtype of ovarian cancer with poor prognosis (Antony et al., 2019). In this field, an important recent finding was the discovery that the list of cell surface receptors crosslinking with the c-MET signaling is constantly growing, highlighting the importance of this pathway for personalized target therapy (Faiella et al., 2022).

### 3.7 Major Findings Relevant to Other Signaling Pathways

Studying gene regulation in response to ROS, and especially hydrogen peroxide, which started more than 2 decades ago (Müller et al., 1997), continues to retain considerable attention since the discovery of H_2_O_2_ sensors, their suggested role in a receptor-initiated redox signaling pathway (Toledano et al., 2004), and the involvement of peroxiredoxins as regulators (Kang et al., 2005). To date, the interconnection between H_2_O_2_ signaling and EMT in cancer is well established (Milton and Konrad, 2022), and the role of H_2_O_2_ producers NADPH oxidase 4 (NOX4), dual oxidase 1 (DUOX1) and DUOX2 in cell motility and metastasis in cancer biology has been reviewed (Meitzler et al., 2019). Regarding NF-κB, it was demonstrated that inhibiting its activity resulted in an almost complete loss of expression of the Twist2 gene (Lehman et al., 2020). The effects of activating the JAK/STAT3 signaling pathway on EMT and the generation of CSCs have also been documented (Jin 2020). The MAPK signaling pathway represents another signaling pathway involved in EMT, which includes ERK1/2, and Bao et al. (2021) recently reported that a member of the erythrocyte membrane protein band 4.1 superfamily, EPB41L5, overexpressed in esophageal cancer, activated the phosphorylation of ERK/p38 signaling pathway components. Two additional reports have shown the involvement of two other proteins in the activation of this pathway, the seven-transmembrane domain-containing 1 (ELTD1) (Sun and Zhong, 2021), and actin gamma 1 (ACTG1) (Xiao et al., 2021). Concerning PI3K/Akt/mTOR signaling, which regulates the EMT of gastric cancer cells, its aberrant phosphorylation was found to be caused by cooperation between CD36 and apolipoprotein C2 (APOC2) (Wang C. et al., 2021). Among the drivers of glioblastoma progression, both this pathway and Wnt represented key regulators of EMT, two interesting targets for developing treatments (Behrooz et al., 2022). Two interesting findings completed the investigations on this pathway, the knockdown of the mitochondrial ribosomal protein MRPL13 that restrained the EMT process in breast cancer cells (Cai M. et al., 2021), and the role played by the YTH N6-methyladenosine RNA binding protein 1 (YTHDF1) in its activation (Luo et al., 2021). Finally, one last additional pathway of interest is represented by p53, through its links with various drivers of EMT discussed in other chapters of this review. A first report described the importance of a chaperone protein of histone H3-H4, the anti-silencing function 1 (ASF1B), which promotes cell proliferation, migration, and invasion through the modulation of the p53-mediated EMT signaling pathway (Wang et al., 2022). Secondly, Pustovalova et al. shed light on the crosstalk between autophagy and this pathway (Pustovalova et al., 2021). Thirdly, Avşar Abdik (2021) demonstrated how the p53 status affected mature adipocyte-mediated proliferation, emphasizing the importance of targeting the tumor microenvironment.

### 3.8 Transcriptional and Translation Factors

Key inducers of EMT are TFs, and after *Snail*, *Slug, Zeb1*, and *Zeb2*, a critical stage in this research field was reached with the discovery of the role of *Twist* in metastasis (Kang and Massagué, 2004). Subsequently, the basic mechanism for the dynamic silencing of *CDH1*, the gene encoding E-cadherin, by all these TFs started to be deciphered, and post-transcriptional modifications emerged as an additional level of regulation, all of which are being reviewed (Peinado et al., 2007). Since then, the list of TFs has been updated, and new findings in the complex regulatory network they form were recently summarized (Debnath et al., 2022). Regarding Snail-1, the involvement of the B-Raf proto-oncogene (BRAF) gene was highlighted, linked with chemoresistance in thyroid cancer (Wieczorek-Szukala and Lewinski, 2021), and the role of epigenetic regulation was emphasized (Dong and Wu, 2021). Even more important, Snail-1 was shown to be involved in tumor immunosuppression by inducing chemokines and immunosuppressive cells into the tumor microenvironment (Tang et al., 2021). Another family of transcription factors of interest is represented by the SRY-related High Mobility Group (HMG)-box (SOX), which regulates different molecular pathways. Their role in cervical cancer was also recently reviewed (Paskeh et al., 2021b). Finally, as reviewed for osteosarcoma, many miRNAs inhibit EMT through the activation of several signaling pathways upstream of *Snail*, *Slug*, *Twist* and *Zeb1* (Yu et al., 2021).

FOX (forkhead box) proteins also play a crucial role in regulating expression of the genes involved in EMT in cancer. As an increasing number of them were discovered in the last 2 decades of the 20th century, they are presently unified in a nomenclature based on sequence conservation. A first group is represented by class O (FOXO), which are downstream effectors of the PI3K/AKT signaling pathway, and within them FOXO3a was found to crosstalk with the Wnt pathway, negatively regulating β-catenin signaling (Liu et al., 2015). Among the other groups, several recent reports have highlighted the functions of FOXC1 (Ray et al., 2021), FOXI3 (Mukherjee et al., 2018), and FOXM1 (Zhang Y. L. et al., 2021) in EMT.

At the level of translation, altered levels and activities of initiation factors are common facts observed in many cancers, and associated with EMT. Within them, one of the most important, factor 4E (eIF4E), can be targeted by two miRNAs, miR-320a or miR-340-5p, leading to inhibition of MMP-3 and MMP-9 (Zhang H. H. et al., 2020). However, the eIF4F translation initiation complex also contains the ATP-dependent DEAD box RNA helicase eIF4A which remodels the 5′-proximal secondary structure for facilitating 40S ribosome recruitment, and a mechanical study revealed that one of the two isoforms, eIF4A1, functioned as an enhancer of EMT in gastric cancer (Gao et al., 2020). Moving forward, these investigations demonstrated that, in pancreatic cells, the downregulation of E-cadherin produced by the overexpression of eiF4A1 occurred through the c-MYC/miR-9 axis (Zhao Y. et al., 2021).

### 3.9 Non-coding RNAs

MicroRNAs were established as regulators in cancers 2 decades ago, and their involvement as posttranscriptional repressors of gene function in EMT in cancers was reviewed in 2007–2008 (Zavadil et al., 2007; Gregory et al., 2008). MicroRNAs (miRNAs), with 22–24 nucleotides, presently represent a part of ncRNAs divided in two groups based on their base length, small ncRNAs with fewer than 200 nucleotides, and long forms (lncRNAs) with diverse sizes ranging from 200 to more than a thousand nucleotides. More recently, circular RNAs (circRNAs) were shown to proceed differently as they prevent the inhibitory effect of miRNAs on gene expression by binding to their complementary sequences (Meyer et al., 2021). This growing, rapidly evolving field of research and its relationships with EMT in cancer have been recently reviewed (Hussen et al., 2021), and the role of the dysregulated expression of lncRNAs in the pathogenesis of aggressive cancers such as pancreatic ductal adenocarcinoma was particularly emphasized (Takahashi et al., 2021). An important point was the interplay observed between lncRNAs and the biogenesis of CSCs (McCabe and Rasmussen, 2021). Finally, another main field of research focuses on the role of a special category of lncRNAs, defined as intergenic, such as MALAT1, predominantly found in nuclear speckles, and which are associated with poor prognosis cancers. Its involvement in metastatic colorectal cancer was also recently reviewed (Uthman et al., 2021).

### 3.10 Tumor Microenvironment

One of the most important EMT driving parameters of the tumor microenvironment is hypoxia. Hypoxia mediated EMT is one of the three modules of Notch-mediated EMT involved in breast carcinogenesis, interacting with cytokine and PI3K/Akt mediated EMT. Hypoxia was shown to elevate the expression of Notch effectors downstream, such as hairy and enhancer of split-1 (HES1) and Hes related family bHLH transcription factor with YRPW motif (HEY1), that in turn upregulate Slug and Snail expression (Kar et al., 2019). Multiple hypoxia-induced EMT markers have been identified, including histone deacetylase 3 (HDAC3), WD repeat domain 5 (WDR5), histone H3 acetylated at lysine 4 (H3K4Ac) at the chromatin level, and two transcription factors, smoothened (SMO) and Gli1 (Lin and Wu, 2020). In this field, Zheng et al. recently reported, in an analysis of 431 gastric cancer samples, its impact on changes in tumor stroma components, as a gene set enrichment analysis revealed that EMT, TGF-β signaling, hypoxia, and angiogenesis gene sets were significantly enriched in CAFs, and linked with a worse prognosis (Zheng H. et al., 2021). Hypoxia and hypoxia-induced ROS are among the most important physiological regulators of de-differentiation and thus have a profound impact on the acquisition of a motile phenotype in cancer cells and the metastatic process (Jensen, 2015).

Another consequence of hypoxia is an increase in the expression of the VEGF, which contributes to angiogenesis by recruiting endothelial cells into hypoxic areas and stimulating their proliferation. In the relationship that links EMT and tumor angiogenesis, the Twist1-Jagged1-kruppel-like factor 4 (KLF4) axis appears to play a crucial role (Chen and Wu, 2016). In normal tissues, endothelial cells that line blood vessels serve as a barrier to the movement of cells into or out the blood. However, although it has long been debated whether or not cancer cells are passively shed into the circulation due to defective vessels, it is now established that cancer cells secrete growth factors and cytokines that produce vascular hyperpermeability and compromise endothelial barrier function, thereby facilitating their transmigration through the vascular wall (Shenoy and Lu, 2016). Another process of microvascularization, which differs from angiogenesis but is also related to EMT, is vasculogenic mimicry (VM), by which highly aggressive tumor cells form vessel-like structures (Kotiyal and Bhattacharya, 2015). Increasing evidence has demonstrated how, under hypoxia and high interstitial fluid pressure, the EMT-inducing TF Twist1 induces CSCs to differentiate into endothelium-like cells expressing the endothelium marker VE-cadherin, promoting ECM remodeling in VM (Sun et al., 2017). Endothelial cells in tumors exhibit remarkable plasticity, as revealed by EndMT, where endothelial cells convert to mesenchymal cells, giving rise to fibroblasts but also bone cells (van Meeteren and ten Dijke, 2012). Recent findings have emphasized the role of enhancers of EndMT that play the role of inflammatory signaling molecules in various cancers, modulating the tumor microenvironment by affecting the immune cell response (Yoshimatsu and Watabe, 2022). Finally, Lin (2020) reported that among endothelial cells making up the lining of tumor vasculature, most are aneuploid tumor-derived endothelial cells (TECs) generated from “cancerization of stromal endothelial cells” and “endothelialization of carcinoma cells,” both being related to EMT, and that share with CTCs the ability to shed into peripheral circulation.

Among stromal components of the tumor microenvironment involved in EMT, a new player was recently identified that could explain the peculiar role of the peritoneal cavity as a privileged location for homing spots for secondary tumors, represented by adipocytes (Mikula-Pietrasik et al., 2018). A plethora of evidence revealed that adipose tissue contributed to cancer progression, and leptin, an adipokine primarily synthesized from adipocytes but also produced by fibroblasts, was shown to be involved in breast cancer (Andò et al., 2014). Cancer-associated adipocytes (CAAs) were generated from primary preadipocytes from mammary fat pads of human breast cancer patients that highly expressed the granulocyte colony-stimulating factor gene (G-CSF), conferring an invasive advantage on triple-negative cancer cells for their progression through EMT *via* STAT3 signaling (Liu et al., 2020). In pancreatic cancer, Takehara et al. (2020) also reported the ability of adipocytes to de-differentiate to CAAs when co-cultured with cancer cells, EMT being induced in the latter via expression of serum amyloid A1 (SAA1), a pro-inflammatory cytokine also expressed in breast cancer cells and TAMs, leading to poorer prognosis in patients.

Over the last 2 years, considerable attention has been given to the role of CAFs in EMT since the pioneering work of Gaggioli who highlighted the fact that epithelial cancer cells could take advantage of the mesenchymal characteristics of CAFs without the need to undergo EMT themselves (Gaggioli 2008). To give just a few of the numerous breakthroughs, CAFs were shown to highly express lysyl oxidase, leading to an increase in matrix stiffness, promoting the EMT process in squamous cell carcinoma cells through the focal adhesion kinase phosphorylation pathway (Zhang J. Y. et al., 2021). The poor prognosis of patients with gastric cancer was explained by a high expression of galectin-1 in the microenvironment, produced by CAFs, promoting invasion of cancer cells via EMT through the TGF-β1/Smad signaling pathway (You et al., 2021). A new mechanism was elucidated for the acquisition of an invasive phenotype of non-neoplastic breast cells, induced by CAFs, involving interleukin 8 (IL-8) and S100A8 (Lim et al., 2021). CAFs isolated from lung adenocarcinoma tumors promoted EMT via production of stromal-derived factor-1 (SDF-1), making it possible to identify C-X-C chemokine receptor type 4 (CXCR4)/β-catenin/peroxisome proliferator-activated receptor gamma (PPARδ) signaling in this process (Wang Y. et al., 2021). TGF-β1-activated CAFs appeared to promote EMT and breast cancer growth by autophagy or overexpression of fibroblast-activating protein-α (Huang et al., 2021). Finally, dynamic crosstalk between CAFs and cancer cells involve transfer of genetic messages such as ncRNA-loaded exosomes, and in this field, a lncRNA was recently characterized as participating in this process (Zhou et al., 2021). Consequently, bearing in mind all these improvements, today CAFs represent a crucial therapeutic target for getting rid of the deleterious effects of EMT in invasive cancers (Es et al., 2021).

Regarding CSCs, Paramanantham et al. (2021) demonstrated how, in the mechanisms accounting for the maintenance and/or induction of EMT and CSCs, the doxorubicin resistance of human breast cancer cells was transferred to parental, sensitive cancer cells through autocrine signaling. To improve our understanding of cellular communication, Acuña et al. showed that one member of the connexin family, Cx46, representing transmembrane proteins forming two different types of ion channels allowing the cytoplasm to communicate with ECM, modulated CSC and EMT properties in breast cancer cells (Acuña et al., 2021). In parallel, the role of T-box genes has also been highlighted, as they were reported to drive or repress EMT in cancer, while some of them positively regulate CSCs, including brachyury (TBXT), TBX3, TBX5, TBX19, and TBX21 (Niu et al., 2022).

The last important stromal component involved in EMT in cancer corresponds to immune cells. Among them, the means used by the three categories of immunosuppressive cells, including regulatory T cells (T_regs_), myeloid-derived suppressor cells (MDSCs) and TAMs, to repress the activity of T cells, were recently reviewed (Taki et al., 2021). Focusing on T_regs_, their expression being increased in hepatocellular carcinoma and associated with tumor metastasis and poor prognosis, Shi et al. found that invasion was promoted by TGF-β1-induced EMT (Shi et al., 2018). Moreover, Kinoshita et al. (2020) also reported that the combined secretion of TGF-β1and interleukin 6 (IL-6) by cancer cells induced a heterogeneity of T_regs_ and/or interleukin 17 (IL-17)-producing T helper 17 cells, favoring the progression of biliary tract cancer cells. Monocytes and monocytic-MDSCs are recruited from blood to the tumor by several cytokines, including CCL2, CCL5 and CSF1, which differentiate into TAMs (Taki et al., 2021). Recent investigations on TAMs have revealed they induce EMT in osteosarcoma cells by activating the COX-2/STAT3 axis (Han et al., 2019). Subsequently, a pan-cancer EMT analysis of 22 cancer types in TCGA datasets highlighted that the first distinctive feature of the EMT-high (mesenchymal) tumors was the enrichment in TAMs (Tiwari et al., 2021). Regarding MDSCs, the distinct role of the different subsets in tumor EMT was reviewed (Cai et al., 2021a), while inducing EMT and metastatic properties in lung cancer cells was found to be promoted by MDSCs through CCL11 and the activation of ERK and AKT signaling (Lin S. et al., 2021). Papadaki and colleagues’ data also showed a clear association between CTCs (bearing EMT or/and CSCs features) and a subset of CD14^+^ CD15^+^ MDSCs in metastatic breast cancer patients (Papadaki et al., 2021).

## 4 Molecular and Cellular Cancer EMT: Targets of Curcuminoids

**FIGURE 4:**
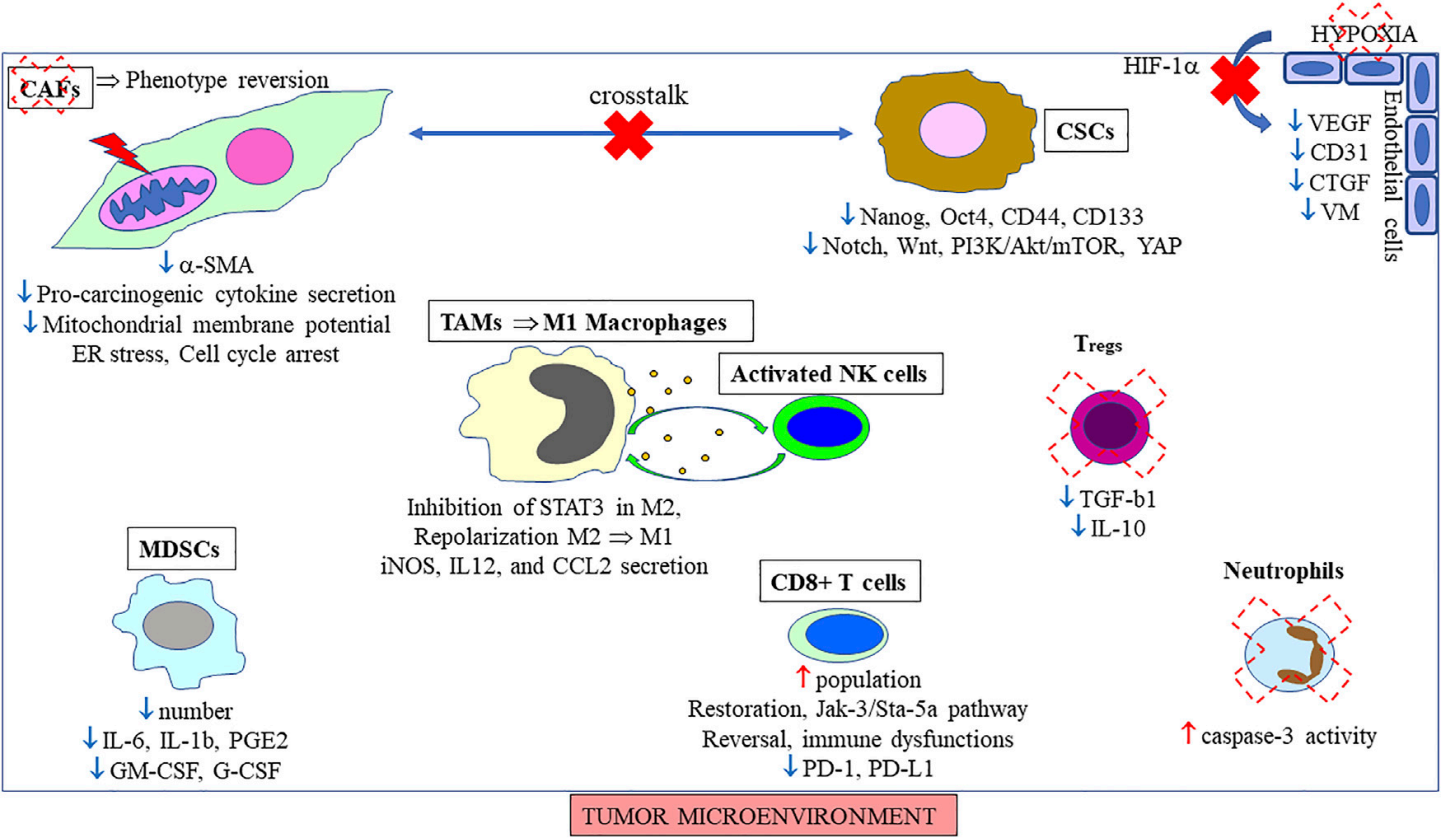
Effects of curcumin/curcuminoids on tumor microenvironment. The main positive (**↑**)/negative (**↓**) reported in the literature are indicated for each component./illustrate crosstalk suppression/reduction, and the red thunderstorm mitochondrial changes.

Like other categories of phytochemicals, curcuminoids are characterized by their ability to simultaneously target multiple signaling pathways, an observation often associated with the concept of “polypharmacology” (Franci et al., 2010). This has been well reviewed in past decades and confirmed more recently in questions relevant to EMT in cancer (Gall Trošelj et al., 2020), where the relationships with CSCs are a growing field of interest (Cianciosi et al., 2018). More precisely, Buhrmann and colleagues have shown that curcumin had the potential to dramatically decrease the crosstalk between CSCs and stromal fibroblasts (Buhrmann et al., 2014) (Figure 4). Additionally, an important aspect of its pharmacological effects relies on inhibition of several pathways important for CSCs, such as NF-κB, PI3K/Akt/mTOR, Notch, Wnt, and Hippo/YAP (Ke et al., 2021), but also of signaling crosstalks involved in chemoresistance (Vinod et al., 2013). Moreover, unlike other Hedgehog inhibitors that produced some adverse effects *in vivo*, the combination of curcumin with chemotherapeutics or targeted agents has represented a new therapeutic strategy with low or no toxicity (Li et al., 2012; Dong et al., 2021). The subcellular targets of 16 curcumin analogs have more specifically been reviewed (Adeluola et al., 2021), while complementary data on EMT potential targets for eight additional analogs are summarized in Table 1. In this field, the targeting of EMT pathways by curcumin analogs such as ST09 produced a significant reduction in tumor growth without any adverse systemic toxicity *in vivo* (Ravindran et al., 2021). Hypoxia and the HIF signaling pathway led to enrichment in CSCs, and curcumin/curcuminoid effects against cancer EMT are mediated through its action on this major tumor microenvironment factor, through a decrease in the expression of miR-21, miR-210, IL-6, HIF-1α, and VEGF (Bao et al., 2012b). Another targeted growth factor involved in tumor angiogenesis (Shen Y. W. et al., 2021) is connective tissue growth factor (CTGF) (Shao et al., 2019). Jiao et al. (2016) has also shown that curcumin mimicked the effects of c-Met inhibitors, blocking c-Met phosphorylation and downstream activation of Akt, mTOR and S6. This blockade of c-Met by curcumin was recently confirmed, inhibiting the increase in vimentin (Onishi et al., 2020), an interesting finding as c-Met engagement is known to activate multiple signal transduction pathways, such as RAS (“Rat sarcoma virus”), PI3K, STAT, Wnt and Notch. To give just one more example of the positive consequences of the effects of curcumin in this specific field of research, Zheng et al. recently reviewed its additional ability to suppress VM in hepatocellular carcinoma (Zheng N. et al., 2021). Regarding CAFs, curcumin downregulated the expression of alpha-smooth muscle actin (α-SMA) and inhibited their secretion of pro-carcinogenic cytokines, such as TGF-β1, MMP2, and stromal cell-derived factor 1 (SDF-1) (Ba et al., 2020). Moreover, the molecular effects of curcumin on CAFs include ROS-mediated ERS through the protein kinase R-like endoplasmic reticulum kinase (PERK)-eIF2α-activating transcription factor 4 (ATF4) axis, and a decrease in mitochondrial membrane potential, leading to apoptosis (Zeng et al., 2020). Finally, in link with Figures 2, 3, the analysis of whole transcriptome alteration in curcumin-treated lung cancer cells also identified ECM receptor genes involved in EMT (Li et al., 2017).

**TABLE 1:** Curcuminoids (curcumin synthetic analogs) and their potential EMT targets.

Analogs	Signaling pathways/Targets	Authors
AC17	-	Adeluola et al. (2021)
ACS-J9	ROS, p53, epigenetic changes	
B19	ROS generation, p38 MAPK	
BS109	-	
CDF	CSCs, miRNAs, NF-κB	
CLEFMA	NF-κB, JNK	
DM-1	-	
EF24, EF31	NF-κB, HIF1α, ROS, angiogenesis	
FLLL12, FLLL31, FLLL32	JAK/STAT, p-AKT	
GO-Y030	NF-κB, Pi3K/AKT, JAK/STAT	
HO-3867, HO-4918	p53, pAKT/STAT3	
PAC	NF-κB, Wnt	
UBS109	NF-κB, Angiogenesis	
BHMC	miRNAs	Yeap et al., 2021
Comp34	lncRNA = NUDT3-AS4	Hao et al. (2020)
WZ26	ROS, JNK	Zhang T. et al. (2019)
WZ35	ROS, p38, Hippo	Wang et al. (2019)
ST09	miRNAs	Ravindran et al. (2021)
GL63	lncRNA = circZNF83/miR-324-5p	Zhao J. a. et al. (2021)
L48H37	JAK/STAT	Lu K. H. et al. (2021)
ZYX02-Na	Autophagy	Zhou G. Z. et al. (2020)

Among immune cells, the populations of two other categories were affected by curcumin treatment, neutrophils through activation of the p38 MAPK pathway (Hu et al., 2005), and T_regs_ *via* downregulation of TGF-β and interleukin-10 (IL-10) in these cells (Bhattacharya et al., 2010) (Figure 4). Conversely, curcumin was found to prevent tumor-induced apoptosis of thymic and circulating CD4+/CD8+ single/double positive T cells through downregulation of Bax while increasing Bcl-2 expression through restoration of the cytokine-dependent Jak-3/Stat-5a signaling pathway (Bhattacharya et al., 2007). Bose et al. (2015) reviewed other additional positive effects produced by this molecule on T cells and relevant to immune surveillance, emphasizing the fact that curcumin restored the population of CD4^+^ and CD8^+^ T cells in the tumor microenvironment, thereby driving the Th2 cytokine bias towards a Th1 type response. More recent investigations have revealed that the mechanisms by which curcumin reinvigorated defective T cells involved a decrease in programmed cell death protein 1 (PD-1) and T-cell immunoglobulin and mucin containing protein-3 (TIM-3) on T_regs_, an increase in the secretion of Interferon gamma (IFN-γ) and Granzyme B, potentiating the effect of CD8^+^ T cells-mediated cancer cell killing (Liu L. et al., 2021). Concerning macrophages, Baidoo et al. (2021) discovered that tumor-promoting M2 macrophages were repolarized into nitric oxide-generating tumoricidal M1 macrophages by curcumin treatment, an action produced by even low, transient levels of this phytochemical *in vivo*. Finally, regarding MDSCs, Bill et al. (2012) found early on those two analogs of curcumin, FLLL32 and FLLL62, inhibited the generation of myeloid-derived suppressor cells *in vitro* via STAT3 phosphorylation. Tu et al. (2012) demonstrated that curcumin induced the differentiation of MDSCs isolated from the spleen, blood, and tumor tissues of mice with gastric cancer toward M1-like phenotype, additionally suppressing the interaction between MDSCs and cancer cells, finally leading to tumor growth suppression. Interestingly, Tian et al. (2021) revealed curcumin suppressed the secretion of granulocyte macrophage colony stimulating factor (GM-CSF) and granulocyte-colony stimulating factor (G-CSF) by MDSCs (Figure 4), two essential factors for their modulation into tumor tissues, with their number also reduced through inactivation of the Toll like receptor 4 (TLR4)/NF-κB signaling pathway.

We have seen above that among the multiple signaling pathways on which curcuminoids act in cancer EMT, NF-κB plays a special role as it ultimately leads to the silencing of many inflammatory cytokines involved in crosstalk between several stromal components of the tumor microenvironment. This point and its connections with other signaling pathways were especially reviewed in breast cancer by Song et al. (2019). Complementary investigations based on transcriptomic profiling revealed curcumin attenuated the upregulation of three NF-κB-regulated chemokines, CXCL8, CXCL1 and CXCL2, associated to oxaliplatin resistance in colorectal cancer cells (Ruiz de Porras et al., 2016). Another crucial target was growth hormone (GH), expression of which is remarkably high in aggressive breast cancer cases compared to healthy breast tissues. Coker-Gurkan et al. (2018) found that the autocrine GH-induced NF-κB signaling triggering this aggressive phenotype via JAK/STAT and Akt/MAPK was prevented by curcumin. NF-κB was identified as an important component in H_2_O_2_/ROS signaling, which regulates survival and proliferation (Saunders et al., 2010) (Figure 3). Subsequently, inhibition of H_2_O_2_-induced ROS production and the ERK/NF-κB pathway with curcumin was confirmed (Cao et al., 2016). EMT in pancreatic cancer cells could also be prevented by curcumin-induced inhibition of the H_2_O_2_/PI3K/Akt/NF-κB axis (Li W. et al., 2018). A curcumin-induced decrease in p-Akt was also observed in glioblastoma (Wang Z. et al., 2020) and colon carcinoma cells (Kaur and Moreau., 2021), and in both cases combined inhibition of the Akt/mTOR signaling pathway was reported (Figure 3). Finally, another consequence of enhanced ROS accumulation was a decrease in mitochondrial membrane potential and cytochrome c release from the mitochondria into the cytosol (He et al., 2016).

Upstream of Akt, downregulation of the MAPK/ERK protein serine kinase MEK2 (MA2K2) was also reported (Chiu et al., 2021), thus, effectively, inhibiting proliferation and inducing apoptosis by curcuminoids in cancer cells can involve the p38 MAPK pathway (He et al., 2021; Su et al., 2021) (Figure 3). Blocking ERK1/2 expression in glioma cells by curcumin was also observed in another study, confirming that the MAPK signaling pathway is a potential target (Wang P. et al., 2021). The potent inhibition of EMT in cancer produced by curcumin can also be explained by the fact that RTKs represent one of the main modes of its action, upstream of the MAPK, PI3K/Akt, JAK/STAT, and NF-κB signaling pathways (Dev et al., 2021). However, among downstream targets, curcuminoids were additionally found to activate p53 (Xu et al., 2020). Mechanistically, Patiño-Morales et al. demonstrated that the cytotoxic effect of curcumin against cervical cancer cells involved promotion of the complex it forms with NAD(P)H quinone dehydrogenase 1 (NQO1), leading to its stabilization, thus avoiding the interaction between p53 and its negative regulator ubiquitin ligase E6-associated protein (Patiño-Morales et al., 2020) (Figure 3).

An interesting field of investigation is also identification of downstream target genes of curcumin/curcuminoids. A first key finding was the discovery that the curcumin treatment of pancreatic carcinoma cells suppressed interleukin enhancer binding factor 2 (ILF2) overexpression, which regulated EMT-associated genes in this type of cancer (Bi et al., 2017). Besides translation factors, downstream of HIF-1α, curcumin markedly affects the expression of EMT TFs such as Twist (Kim et al., 2013), and Snail (Lee et al., 2015). Finally, Bagui et al. (2018) reported that triple-negative breast cancer cells, which exhibited aberrant activity of EMT, were characterized by their reduced expression of zinc finger E-box-binding homeobox 1 (ZEB1) after treatment with a nanoformulation of curcumin (Figure 3).

Investigations into the main signaling pathways involved in EMT and detailed above led to identifying some important additional molecular targets affected by curcuminoids in cancer cells. Concerning the TGF-β pathway, a key observation was the decreased phosphorylation of Smad2 and Smad3 produced by curcumin (Zhang et al., 2016). Interestingly, IL-6 secretion induced by Smad2 can be severely impeded by curcumin, attesting of its inhibitory effect on AP-1 (Park et al., 2003). In colon cancer, the effects of curcumin on the inhibition of TGF-β1-regulated activation were also reviewed, emphasizing the fact that beside Smad2 and MMP-9, ERK1/2 and p38 were also affected in a dose- and time-dependent manner (Ramamoorthi and Sivalingam, 2014) (Figure 3). Regarding the Notch signaling pathway, the treatment of cholangiocarcinoma cells with curcumin led to a reduction in Notch1, HEY1, and survivin (Koprowski et al., 2015) (Figure 3). Subsequently, this inhibitory effect of curcumin on Notch1 was confirmed *in vivo* on xenografts of human cervical cancer in nude mice (He et al., 2018), with an impact on NF-κB as the Notch pathway controls its activation (He et al., 2019). Another main pathway involved in the EMT process is Wnt/β-catenin. Vallée et al. reviewed the mechanisms by which curcumin inhibits this pathway, emphasizing the fact that it acted as peroxisome proliferator-activated receptor gamma agonists (Vallée et al., 2019). Within this pathway, a crucial downstream gene targeted by curcumin is Axin2, which acts as a negative feedback regulator, promoting β-catenin degradation (Hao et al., 2021). Furthermore, the combination of curcumin and N-n-butyl haloperidol iodide treatment revealed a downregulation of the enhancer of the zeste homolog 2 (EZH2) in hepatocarcinoma cells, an epigenetic regulator interacting with β-catenin (Khan et al., 2021). Lu et al. (2020) also found that curcumin treatment upregulated the expression level of Tet methylcytosine dioxygenase 1 (TET1), another epigenetic regulator, which in turn upregulated Naked cuticle homolog 2 (NKD2), an inhibitor of Wnt signaling (Figure 3). Finally, an interesting observation was the inhibition produced by curcumin on both β-catenin and Gli1, two members of the Wnt and Hedgehog EMT signaling pathways, respectively, which tended to demonstrate the existence of a physical interaction between them (Zhang X. et al., 2020). Wnt and Shh pathways are crucial for maintaining the stemness of CSCs, and Li X et al. (2018) reported previously that additional CSC markers such as CD44, aldehyde dehydrogenase 1 family, member A1 (ALDH1A1), Nanog and Oct4 are decreased by curcumin in breast cancer cells. Alterations in Shh signaling proteins, such as Gli1, Gli2, Sufu and NF-κB65, were also documented in pancreatic cancer treated with a nanoformulation of curcumin (Khan et al., 2019). The observation that curcumin indeed targeted Gli1 in triple negative breast cancer was also recently confirmed by another team (Li M. et al., 2022) (Figure 3). Regarding the Hippo signaling pathway, Ye et al. reported that the curcuminoid CL-6 induced Lats and phosphorylation of YAP in two gastric cancer cell lines, thereby inactivating YAP (Ye et al., 2019). Mechanistically, another important finding was the proteasome degradation of another downstream protein of this pathway by curcumin, the WW domain containing transcription regulator (WWTR1, also named TAZ), following its phosphorylation (Zheng et al., 2020) (Figure 3). The last important EMT signaling pathway involves TNF-α. Curcumin’s action on this pathway *in vitro* and *in vivo* was extensively reviewed about 1 decade ago (Aggarwal et al., 2013). On the established basis of curcumin’s action on NF-κB (detailed above), Xia et al. (2016) revealed that the curcumin derivative W346 inhibited the NF-κB activation induced by TNF-α by suppressing IKK phosphorylation and inhibiting degradation of IκB-α in human gastric cancer cells (Figure 3).

## 5 Non-coding RNAs as New Targets of Curcuminoids

The ability of curcuminoids to target multiple signaling pathways involved in EMT in cancer has led researchers to investigate its action on ncRNAs (Toden et al., 2015), a rapidly growing research field as a 5-times increase was observed in the number of relevant articles between 2008-2016 and 2017-2022 on the PubMed database. Moreover, a special focus on EMT was observed during the two last years. Together with DNA methylation changes induced by curcumin, the first functional genomic studies allowed to profile several miRNAs of interest (Huminiecki et al., 2017). The list was recently updated, identifying miR-7, miR-17-5p, miR-20a, miR-21, miR-22, miR-27a, miR-29b, miR-33b, miR-34a, miR-34c, miR-49, miR-101, miR-141, miR-185, miR-200a, miR-200b, miR-200c, and miR-429as potential targets (Ashrafizadeh et al., 2020b; Akbari et al., 2021). Among new insights, microarray and qPCR analysis showed that miRNA regulation by curcumin analogs such as BHMC (Table 1) involved both upregulation of miR-3195 and miR-30a-3p, which target the two EMT drivers VEGF and SNAIL, and downregulation of miR-6813-5p and miR-6132 (Yeap et al., 2021).

Linked to the TGF-β signaling pathway, insights into the role of curcumin on miR-19 revealed that it increased the expression of transcription factor Tap63α, which inhibits the lung cancer EMT induced by tobacco smoke by transcriptionally suppressing miR-19 (Xie et al., 2021). Concerning miR-21, linked to the Wnt pathway, curcumin upregulated the expression of a membrane-anchored MMP inhibitor, the reversion-inducing cysteine-rich protein with kazal motifs (RECK), which is a target of miR-21 (Zhou L. et al., 2020). Subsequently, Chen L. et al. (2020) identified the Von Hippel-Lindau (VHL) as another direct target of miR-21, curcumin exerting its anti-proliferation, anti-migration, anti-invasion through miR-21/VHL axis. The last 3 years were also characterized by the demonstration of the impact of curcuminoids on some newly identified miRNAs. Curcumin promoted tumor-suppressive autophagy in triple-negative breast cancer cells CSCs, regulated by miR-181a (Park et al., 2022). The upregulation of miR-192 observed in cisplatin-resistant lung cancer cells was reversed by curcumin-induced inhibition of the NF-κB signaling pathway (Li Y. et al., 2022). The mi-RNA-transcriptome profiling of ovarian cancer cells treated with the curcumin analog ST09 (Table 1) also confirmed the downregulation of EMT pathways, identifying the miR-199a-5p/DDR1 axis as an important target (Ravindran et al., 2021). Finally, among miRNAs that play a tumor-suppressing role, Wang et al. reported the upregulation of miR-206 in curcumin-treated lung cancer cells (Wang N. et al., 2020), the inhibition of the PI3K/AKT/mTOR signaling pathway suppressing migration and invasion.

In the field of lncRNAs, the modulation of their expression by curcumin in various cancers has recently been reviewed, including AB073614, AK294004, ANRIL, BC200, CCAT2, FAL1, GAS5, H19, HOTAIR, KCNQ1OT1, LINC01021, Linc-Pint, LSINCT5, MALAT1, MEG3, NB5R2, PANDAR, PVT1, ROR, UCA1, and XIST (Gowhari Shabgah et al., 2021). H19, especially, which has been the subject of about 60 recent publications linked to EMT in cancer, was found to be attenuated in tamoxifen-resistant breast cancer cells after curcumin treatment (Cai et al., 2021b). Among new findings, curcumin’s potential role in the treatment of resistant acute myeloid leukemia was shown to involve inhibition of the HOTAIR/miR-20a-5p/WT1 axis (Liu J. M. et al., 2021). Moreover, the inhibition of Warburg effect produced by curcumin in invasive thyroid cancer cells, linked to a reduction of the expression of another lncRNA, LINC00691, appeared to involve the Akt signaling pathway (Li Z. et al., 2022). Hao et al. (2020) revealed that a new curcumin analog Comp34 decreased the expression of a newly identified oncogenic lncRNA, NUDT3-AS4, in triple-negative breast cancer cells, leading to the dissociation of the complex formed with miR-99s and finally to the degradation of *AKT1*/*mTOR* mRNA. The effect of curcumin analog EF24 on the overexpression of another lncRNA, the human leukocyte antigen complex group 11 (HCG11), has just started to be documented too (Duan et al., 2022).

Using a high-throughput microarray, Yang et al. (2020) showed how curcumin regulated the circRNA network, establishing a link with radiosensitization of nasopharyngeal carcinoma cells. As CircRNAs usually sponge miRNAs, recent investigations on the effects produced by curcuminoids have added even more complexity to this huge regulation network. These included the effect of the curcumin analog GL63 (Table 1) on the circZNF83/miR-324-5p/CDK16 axis (Zhao J. a. et al., 2021), and of curcumin on the PLEKHM3/miR-320a/SMG1 axis (Sun and Fang, 2021), circFOXP1/miR-520a-5p/SLC7A11 (Wang W. et al., 2021), and circ-FNDC3B/miR-138-5p/IGF2 axis (Xue et al., 2021).

## 6 New Insights From Malignant Mesothelioma Studies

EMT was identified early as an important parameter involved in the pathogenesis of MM (Kamp, 2009). This is related to the fact that mesothelial cells, while deriving from the mesoderm through EMT during their differentiation, exhibit many characteristics of epithelial cells (Batra and Antony, 2015). Ramundo et al. (2021b) in their review of the importance of EMT in the pathogenesis of this devastating cancer, have recently emphasized its crucial role through the involvement of TGF-β, tumor microenvironment crosstalk, immunosuppression, and oxidative stress, linked to previous findings related to hypoxia and HIF-1α (Kim et al., 2018). In our identification of MM biomarkers associated with the highest invasiveness, the highest expression level of fibroblast growth factor 2 (FGF2) specifically observed in the rat experimental tumor M5-T1, in comparison with the two less invasive F5-T1 and F4-T2 (Nader et al., 2018), agreed well with the reported important role of this growth factor (Schelch et al., 2018).

Regarding the role that curcumin treatment plays in decreasing the high abundance, or high expression level of cancer EMT biomarkers, previous observations pointed to two important targets, fibronectin, and vimentin. Linked to activation of the TGF-β1 pathway, fibronectin helps guide cancer cells to migrate, and high expression is also observed in cells with CSC capabilities (Cervantes-Arias et al., 2013). The secretion of oncofetal fibronectin by CAFs mediates the development of an invasive phenotype in cancer cells through reorganization of the ECM (Berndt et al., 2015). The expression of fibronectin was also increased when colon cancer cells were co-cultured with mesenchymal stem cells (MSCs), being localized at the edges of cancer clusters where cancer cells directly came into contact with MSCs (Takigawa et al., 2017). Moreover, fibronectin is a useful biomarker of EMT-induced chemo-resistance in hepatocellular carcinoma cells (Karaosmanoğlu et al., 2018). The role of CAF-secreted fibronectin as an extracellular driver of cancer progression was emphasized, serving as a scaffold for invasion, promoting cell growth, and serving as a nidus for new vessel formation (Rick et al., 2019). Interestingly, the parallel increase we observed between fibronectin and S100A4 during mesothelial cell tumorigenesis (Nader et al., 2020), and the dramatic increase associated with the highest tumor invasiveness (Nader et al., 2018), resonate with findings reporting cooperation between fibronectin and S100 proteins in shaping the pre-metastatic niche in lung, liver, and bone (Paolillo and Schinelli, 2019). In the context of the metastatic colonization of the liver by the most invasive experimental rat MM cell line, M5-T1, we found fibronectin and vimentin, showing increased abundance compared to normal rats, which was completely reversed in curcumin-treated rats (Pouliquen et al., 2020). In the same study, a tendency toward the same evolution was observed for S100A4, while two other proteins in the S100 family, encoded by the *S100a6* and *S100a11* genes, exhibited a similar increase in untreated tumor-bearing rats, which was completely reversed upon curcumin treatment. Subsequently, another investigation conducted on lymphoid organs collected from the same three groups of rats revealed that the decrease observed in fibronectin and S100A4 levels in the spleen was among the list of biomarkers of therapeutic efficacy identified in curcumin-treated rats (Pouliquen et al., 2021).

Our data obtained using MM as experimental tumor models involving EMT, and the observations we made on curcumin-treated rats are consistent with previous reports documenting the effects of curcuminoids on vimentin and fibronectin (Figure 3). In the first case, curcumin’s ability to inhibit the increase in the levels of vimentin produced by hepatocyte growth factor (HGF) involved a downregulation of the expression of phosphorylated c-Met (Onishi et al., 2020). Evidence for a downregulation of vimentin produced by curcumin in a concentration-dependent manner, in parallel with the transcription factors Snail1 and Twist, has previously been described (Chen T. et al., 2020). Li et al. (2020) have also reported another mechanism by which a curcumin-induced decrease in vimentin expression was comparable with the effect of IL-6-neutralizing antibody treatment on pancreatic cancer cells, suggesting it interfered with tumor-stromal crosstalk under hypoxic conditions (Figure 3). Another line of evidence in favor of this latter mechanism came from our observation of a parallel decrease in vimentin and IL-6 expression in residual MM tumors from curcumin-treated rats relative to untreated rats (Pouliquen et al., 2017). In the second case, curcumin was shown to inhibit adhesion and haptotactic migration of hepatocellular carcinoma cells to fibronectin (Ohashi et al., 2003), and to reduce the secretion of fibronectin by leiomyoma cells (Malik et al., 2009). However, these effects were not limited to curcumin as other synthetic curcuminoids exhibited inhibitory effects against fibronectin, an important player in tumor angiogenesis (Shimazu et al., 2018). Finally, our results obtained on MM agreed with the findings of two studies reporting the effects of curcumin treatment on the downregulation of both fibronectin and vimentin in breast cancer cell lines (Gallardo and Calaf, 2016), and the inhibition of stem cell gene expression and mammosphere formation (Chenxia et al., 2019).

In our studies on the effects of curcumin against the metastatic process of an aggressive model of rat MM, we found several members of the S100 protein family were affected, and to the best of our knowledge these were the first reports showing the impact of this phytochemical simultaneously on S100A4, S100A6, S100A8 and S100A11 (Pouliquen et al., 2020; 2020b; 2021). The involvement of S100A4 in EMT and MM has been previously documented in a large collection of 109 tumor specimens from patients, showing its specific high expression in the sarcomatoid histological subtype, together with vimentin and ZEB1, linked to aggressive features (Fassina et al., 2012). Interestingly, the combined decrease in S100A4 and fibronectin levels we specifically observed in the spleen of curcumin-treated rats (Pouliquen et al., 2021) found an echo in the findings of one previous study reporting a connection between Hedgehog signaling and S100a4 regulation in pancreatic cancer cells (Xu et al., 2014). Together with S100A4, S100A6 is the most widely documented protein in the S100 family and is a potential biomarker of tumor invasiveness in proteomics in MM and other cancers (Pouliquen et al., 2020b), and their combined involvement in breast cancer cell proliferation and motility was recognized early (Fang et al., 2009). Before our investigations, S100A6 in MM had never been documented, however, high levels of both annexin A2 and S100A6 have been associated with poor prognosis in gastric cancer (Zhang et al., 2012), while high serum levels of S100A6 correlated with lymph node metastasis and TNM stage (Zhang et al., 2014). High S100A6 levels in clear cell renal cell carcinoma have also been reported as predicting outcomes in patients (Lyu et al., 2015). However, our data fits well with the findings on another cancer type by Khoontawad et al., who revealed that curcumin-fed hamsters with an experimental cholangiocarcinoma presented a downregulation of both S100A6 and vimentin (Khoontawad et al., 2018). Finally, regarding S100A11, on which the effect of curcumin has never been investigated so far, the first studies on its implication in EMT in cancer revealed this protein was present in a list of 19 pseudopod-specific proteins common to six metastatic human tumor cell lines (Shankar et al., 2010). Subsequently, the LASP1-S100A11 axis was found to modulate TGFβ/Smad signaling in EMT (Niu et al., 2016). Zhang and colleagues confirmed that point, showing that S100A11 promoted accumulation of TGF-β1 expression and upregulation of p-Smad2 and 3 (Zhang et al., 2018), while activation of the p38/MAPK pathway was also involved in its action (Zhang et al., 2019). Over the last 3 years, additional insights into the multiple implications of this protein in the EMT process have been provided, such as its interaction with annexin A2 (Tu et al., 2019), its contribution to higher stiffness-induced mesenchymal shifts through its membrane translocation (Dong et al., 2019), and its participation in multiple signaling pathways, including MAPK3 (ERK1), phosphatidylinositol-4,5-bisphosphate 3-kinase catalytic subunit alpha (PIK3CA), HGF/MET, cyclic adenosine monophosphate response element binding protein (CREBBP) and MMP9 (Cui et al., 2021). Finally, the decrease in S100A11 abundance produced by curcumin treatment in our studies *in vivo* may open up interesting prospects as the only previous published works on this protein in MM reported active secretion of S100A11 by mesothelioma cells *in vitro* (Saho et al., 2016), while its neutralization by an anti-S100A11 antibody inhibited the proliferation of pleural MM cells *in vitro* and *in vivo* (Sato et al., 2018).

## 7 Conclusion

Curcuminoids are interesting molecules both for basic science and translational studies on EMT and cancer invasiveness because they have the potential to simultaneously target multiple signaling pathways. Additionally, they can also interfere with crosstalk between components of the tumor microenvironment. These specificities are related to their ability to directly bind diverse proteins with high affinity where the combination of two hydrophobic phenyl rings with a flexible methylene bridge plays a crucial role, as evidenced by structure-activity relationships and docking studies. An important unsolved question concerns the new technologies and methodologies that may provide insights on the time- and dose-dependency of their effects in the future to fully understand the mechanisms of action of these molecules. Moreover, it is also crucial to distinguish between the primary molecular targets and downstream events. The challenge is further complicated by the fact that depending on the cancer localization, histological subtype and grade/immune status of the host, the patterns of aberrant signaling and microenvironment composition may differ considerably. Although the development of network analyses may help improve our understanding of their complex pharmacology, making it possible to decipher how signaling networks are organized, dynamic modeling also appears necessary for determining changes in time and space. Finally, one more exciting research field concerns the question of biologically active curcuminoid metabolites which may contribute to the overall beneficial pharmacological effects observed *in vivo* against the deleterious implication of EMT in invasive cancers.

## References

[B1] Acuña R. A. Varas-Godoy M. Herrera-Sepulveda D. Retamal M. A. (2021). Connexin46 Expression Enhances Cancer Stem Cell and Epithelial-To-Mesenchymal Transition Characteristics of Human Breast Cancer MCF-7 Cells. Int. J. Mol. Sci. 22, 12604. 10.3390/ijms222212604 34830485 PMC8624448

[B2] Adeluola A. Zulfiker A. H. M. Brazeau D. Amin A. R. M. R. (2021). Perspectives for Synthetic Curcumins in Chemoprevention and Treatment of Cancer: an Update with Promising Analogues. Eur. J. Pharmacol. 906, 174266. 10.1016/j.ejphar.2021.174266 34146588 PMC8286352

[B3] Aggarwal B. B. Gupta S. C. Sung B. (2013). Curcumin: an Orally Bioavailable Blocker of TNF and Other Pro-inflammatory Biomarkers. Br. J. Pharmacol. 169, 1672–1692. 10.1111/bph.12131 23425071 PMC3753829

[B4] Ahmad A. Sakr W. A. Rahman K. M. (2012). Novel Targets for Detection of Cancer and Their Modulation by Chemopreventive Natural Compounds. Front. Biosci. (Elite Ed.) 4 (1), 410–425. 10.2741/388 22201883

[B5] Akbari A. Sedaghat M. Heshmati J. Tabaeian S. P. Dehghani S. Pizarro A. B. (2021). Molecular Mechanisms Underlying Curcumin-Mediated microRNA Regulation in Carcinogenesis, Focused on Gastrointestinal Cancers. Biomed. Pharmacother. 141, 111849. 10.1016/j.biopha.2021.111849 34214729

[B6] Andò S. Barone I. Giordano C. Bonofiglio D. Catalano S. (2014). The Multifaceted Mechanism of Leptin Signaling within Tumor Microenvironment in Driving Breast Cancer Growth and Progression. Front. Oncol. 4, 340. 10.3389/fonc.2014.00340 25505738 PMC4245002

[B339] Antony J. Thiery J. P. Huang R. Y-J. (2019). Epithelial-to-mesenchymal transition: lessons from development, insights into cancer and the potential of EMT-subtype based therapeutic intervention. Phys. Biol. 16 (4), 041004. 10.1088/1478-3975/ab157a 30939460

[B7] Ashrafizadeh M. Zarrabi A. Hashemi F. Zabolian A. Saleki H. Bagherian M. (2020). Polychemotherapy with Curcumin and Doxorubicin via Biological Nanoplatforms: Enhancing Antitumor Activity. Pharmaceutics 12 (11), 1084. 10.3390/pharmaceutics12111084 PMC7697177 33187385

[B8] Ashrafizadeh M. Zarrabi A. Hashemipour M. Vosough M. Najafi M. Shahinozzaman M. (2020b). Sensing the Scent of Death: Modulation of microRNAs by Curcumin in Gastrointestinal Cancers. Pharmacol. Res. 160, 105199. 10.1016/j.phrs.2020.105199 32942019

[B9] Asif P. J. Longobardi C. Hahne M. Medema J. P. (2021). The Role of Cancer-Associated Fibroblasts in Cancer Invasion and Metastasis. Cancers 13, 4720. 10.3390/cancers13184720 34572947 PMC8472587

[B10] Astudillo P. (2021). An Emergent Wnt5a/YAP/TAZ Regulatory Circuit and its Possible Role in Cancer. Semin. Cell Dev. Biol. 125, 45–54. 10.1016/j.semcdb.2021.10.001 34764023

[B11] Avila-Carrasco L. Majano P. Sánchez-Toméro J. A. Selgas R. López-Cabrera M. Aguilera A. (2019). Natural Plants Compounds as Modulators of Epithelial-To-Mesenchymal Transition. Front. Pharmacol. 10, 715. 10.3389/fphar.2019.00715 31417401 PMC6682706

[B12] Avşar Abdik E. (2021). Differentiated Pre-adipocytes Promote Proliferation, Migration and Epithelial-Mesenchymal Transition in Breast Cancer Cells of Different P53 Status. Mol. Biol. Rep. 48, 5187–5198. 10.1007/s11033-021-06521-8 34213707

[B13] Azad T. Rezaei R. Surendran A. Singaravelu R. Boulton S. Dave J. (2020). Hippo Signaling Pathway as a Central Mediator of Receptors Tyrosine Kinases (RTKs) in Tumorigenesis. Cancers 12, 2042. 10.3390/cancers12082042 PMC7463967 32722184

[B14] Ba P. Xu M. Yu M. Li L. Duan X. Lv S. (2020). Curcumin Suppresses the Proliferation and Tumorigenicity of Cal27 by Modulating Cancer-Associated Fibroblasts of TSCC. Oral Dis. 26, 1375–1383. 10.1111/odi.13306 32060973

[B15] Bagui N. Bakhshinejad B. Keshavarz R. Babashah S. Sadeghizadeh M. (2018). Dendrosomal Nanocurcumin and Exogenous P53 Can Cat Synergistically to Elicit Anticancer Effects on Breast Cancer Cells. Gene 670, 55–62. 10.1016/j.gene.2018.05.025 29753810

[B16] Bahrami A. Majeed M. Sahebkar A. (2019). Curcumin: a Potent Agent to Reverse Epithelial-To-Mesenchymal Transition. Cell. Oncol. 42, 405–421. 10.1007/s13402-019-00442-2 PMC12994310 30980365

[B17] Bai Y. Sha J. Kanno T. (2020). The Role of Carcinogenesis-Related Biomarkers in the Wnt Pathway and Their Effects on Epithelial-Mesenchymal Transition (EMT) in Oral Squamous Cell Carcinoma. Cancers 12, 555. 10.3390/cancers12030555 PMC7139589 32121061

[B18] Bai L. Sun W. Han Z. Tang H. (2021). CircSND1 Regulated by TNF-α Promotes the Migration and Invasion of Cervical Cancer Cells. Cancer Manag. Res. 13, 259–275. 10.2147/CMAR.S289032 33469369 PMC7811455

[B19] Baidoo J. N. E. Mukherjee S. Kashfi K. Banerjee P. (2021). A New Perspective on Cancer Therapy: Changing the Treated Path? Int. J. Mol. Sci. 22, 9836. 10.3390/ijms22189836 34575998 PMC8466953

[B20] Balkwill F. (2009). Tumour Necrosis Factor and Cancer. Nat. Rev. Cancer 9, 361–371. 10.1038/nrc2628 19343034

[B21] Bao B. Ahmad A. Li Y. Azmi A. S. Ali S. Banerjee S. (2012). Targeting CSCs within the Tumor Microenvironment for Cancer Therapy: a Potential Role of Mesenchymal Stem Cells. Expert Opin. Ther. Targets 16 (10), 1041–1054. 10.1517/14728222.2012.714774 22877147

[B22] Bao B. Ali S. Ahmad A. Azmi A. S. Li Y. Banerjee S. (2012b). Hypoxia-induced Aggressiveness of Pancreatic Cancer Cells Is Due to Increased Expression of VEGF, IL-6 and miR-21, Which Can Be Attenuated by CDF Treatment. PLoS One 7 (12), e50165. 10.1371/journal.pone.0050165 23272057 PMC3521759

[B23] Bao S. Ji Z. Shi M. Liu X. Y. (2021). EPB41L5 Promotes EMT through the ERK/p38 MAPK Signaling in Esophageal Squamous Cell Carcinoma. Pathol. Res. Pract. 228, 153682. 10.1016/j.prp.2021.153682 34784520

[B24] Baran B. Bechyne I. Siedlar M. Szpak K. Mytar B. Sroka J. (2009). Blood Monocytes Stimulate Migration of Human Pancreatic Carcinoma Cells *In Vitro*: the Role of Tumour Necrosis Factor – Alpha. Eur. J. Cell Biol. 88, 743–752. 10.1016/j.ejcb.2009.08.002 19782426

[B25] Batra H. Antony V. B. (2015). Pleural Mesothelial Cells in Pleural and Lung Diseases. J. Thorac. Dis. 7 (6), 964–980. 10.3978/j.issn.2072-1439.2015.02.19 26150910 PMC4466423

[B26] Behrooz A. B. Talaie Z. Jusheghani F. Los M. J. Klonisch T. Ghavami S. (2022). Wnt and PI3K/Akt/mTOR Survival Pathways as Therapeutic Targets in Glioblastoma. Int. J. Mol. Sci. 23, 1353. 10.3390/ijms23031353 35163279 PMC8836096

[B27] Bera A. Lewis S. M. (2020). Regulation of Epithelial-To-Mesenchymal Transition by Alternative Translation Initiation Mechanisms and its Implications for Cancer Metastasis. Int. J. Mol. Sci. 21, 4075. 10.3390/ijms21114075 PMC7312463 32517298

[B28] Berndt A. Richter P. Kosmehl H. Franz M. (2015). Tenascin-C and Carcinoma Cell Invasion in Oral and Urinary Bladder Cancer. Cell adh. Migr. 9 (1-2), 105–111. 10.1080/19336918.2015.1005463 25793577 PMC4422813

[B29] Bhatt A. B. Patel S. Matossian M. D. Ucar D. A. Miele L. Burow M. E. (2021). Molecular Mechanisms of Epithelial to Mesenchymal Transition Regulated by ERK5 Signaling. Biomolecules 11 (2), 183. 10.3390/biom11020183 33572742 PMC7911413

[B30] Bhattacharya S. Mandal D. Saha B. Sen G. S. Das T. Sa G. (2007). Curcumin Prevents Tumor-Induced T Cell Apoptosis through Stat-5a-Mediated Bcl-2 Induction. J. Biol. Chem. 282 (22), 15954–15964. 10.1074/jbc.M608189200 17392282

[B31] Bhattacharya S. Hossain D. M. S. Mohanty S. Sen G. S. Chattopadhyay S. Banerjee S. (2010). Curcumin Reverses T Cell-Mediated Adaptative Immune Dysfunctions in Tumor-Bearing Hosts. Cell. Mol. Immunol. 7, 306–315. 10.1038/cmi.2010.11 20305684 PMC4003225

[B32] Bi Y. Shen W. Min M. Liu Y. (2017). MicroRNA-7 Functions as a Tumor-Suppressor Gene by Regulating ILF2 in Pancreatic Carcinoma. Int. J. Mol. Med. 39, 900–906. 10.3892/ijmm.2017.2894 28259961 PMC5360436

[B33] Bill M. A. Nicholas C. Mace T. A. Etter J. P. Li C. Schwartz E. B. (2012). Structurally Modified Curcumin Analogs Inhibit STAT3 Phosphorylation and Promote Apoptosis of Human Renal Cell Carcinoma and Melanoma Cell Lines. PLoS One 7 (8), e40724. 10.1371/journal.pone.0040724 22899991 PMC3416819

[B34] Böhrnsen F. Holzenburg J. Godek F. Kauffmann P. Moser N. Schliephake H. (2020). Influence of Tumour Necrosis Factor Alpha on Epithelial-Mesenchymal Transition of Oral Cancer Cells in Co-culture with Mesenchymal Stromal Cells. Int. J. Oral Maxillofac. Surg. 49, 157–165. 10.1016/j.ijom.2019.06.001 31345665

[B35] Bornes L. Belthier G. van Rheenen J. (2021). Epithelial-to-mesenchymal Transition in the Light of Plasticity and Hybrid E/M States. J. Clin. Med. 10 (11), 2403. 10.3390/jcm10112403 34072345 PMC8197992

[B36] Bose S. Panda A. K. Mukherjee S. Sa G. (2015). Curcumin and Tumor Immune-Editing: Resurrecting the Immune System. Cell Div. 10, 6. 10.1186/s13008-015-0012-z 26464579 PMC4603973

[B37] Boyer B. Thiery J. P. (1993). Epithelium-mesenchyme Interconversion as Example of Epithelial Plasticity. APMIS 101, 257–268. 10.1111/j.1699-0463.1993.tb00109.x 8323734

[B38] Boyer B. Vallés A. M. Edme N. (2000). Induction and Regulation of Epithelial-Mesenchymal Transitions. Biochem. Pharmacol. 60, 1091–1099. 10.1016/s0006-2952(00)00427-5 11007946

[B39] Brabletz T. Kalluri R. Nieto M. A. Weinberg R. A. (2018). EMT in Cancer. Nat. Rev. Cancer 18 (2), 128–134. 10.1038/nrc.2017.118 29326430

[B40] Brabletz S. Schuhwerk H. Brabletz T. Stemmler M. P. (2021). Dynamic EMT: a Multi-Tool for Tumor Progression. EMBO J. 40 (18), e108647. 10.15252/embj.2021108647 34459003 PMC8441439

[B341] Buhrmann C. Kraehe P. Lueders C. Shayan P. Goel A. Shakibaei M. (2014). Curcumin suppresses crosstalk between colon cancer stem cells and stromal fibroblasts in the tumor microenvironment: potential role of EMT. PLoS One 9 (9), e107514. 10.1371/journal.pone.0107514 25238234 PMC4169561

[B41] Buyuk B. Jin S. Ye K. (2021). Epithelial-to-mesenchymal Transition Signaling Pathways Responsible for Breast Cancer Metastasis. Cell. Mol. Bioeng. 15 (1), 1–13. 10.1007/s12195-021-00694-9 35096183 PMC8761190

[B42] Cai J. Cui Y. Yang J. Wang S. (2021a). Epithelial-mesenchymal Transition: when Tumor Cells Meet Myeloid-Derived Suppressor Cells. Biochim. Biophys. Acta Rev. Cancer 1876, 188564. 10.1016/j.bbcan.2021.188564 33974950

[B43] Cai J. Sun H. Zheng B. Xie M. Xu C. Zhang G. (2021b). Curcumin Attenuates lncRNA H19-Induced Epithelial-Mesenchymal Transition in Tamoxifen-Resistant Breast Cancer Cells. Mol. Med. Rep. 23, 13. 10.3892/mmr.2020.11651 33179087 PMC7673326

[B44] Cai M. Li H. Chen R. Zhou X. (2021). MRPLI3 Promotes Tumor Cell Proliferation, Migration and EMT Process in Breast Cancer through the PI3K-AKT-mTOR Pathway. Cancer Manag. Res. 13, 2009–2024. 10.2147/cmar.s296038 33658859 PMC7920513

[B45] Cao L. Liu J. Zhang L. Xiao X. Li W. (2016). Curcumin Inhibits H_2_O_2_-Induced Invasion and Migration of Human Pancreatic Cancer via Suppression of the ERK/NF-κB Pathway. Oncol. Rep. 36, 2245–2251. 10.3892/or.2016.5044 27572503

[B46] Cervantes-Arias A. Pang L. Y. Argyle D. J. (2013). Epithelial-mesenchymal Transition as a Fundamental Mechanism Underlying the Cancer Phenotype. Vet. Comp. Oncol. 11 (3), 169–184. 10.1111/j.1476-5829.2011.00313.x 22404947

[B47] Chattopadhyay I. Ambati R. Gundamaraju R. (2021). Exploring the Crosstalk between Inflammation and Epithelial-Mesenchymal Transition in Cancer. Mediat. Inflamm. 2021, 9918379. 10.1155/2021/9918379 PMC8219436 34220337

[B48] Chen H. -F. Wu K. -J. (2016). Endothelial Transdifferentiation of Tumor Cells Triggered by the Twist1-Jagged-KLF4 axis: Relationship between Cancer Stemness and Angiogenesis. Stem Cells Int. 2016, 6439864. 26823670 10.1155/2016/6439864 PMC4707371

[B49] Chen H. Yang D. Wang Y. Tao H. Luo Y. Wu A. (2022). Activation of the Hedgehog Pathway Mediates Resistance to Epidermal Growth Factor Receptor Inhibitors in Non-small Cell Lung Cancer. J. Cancer 13, 987–997. 10.7150/jca.63410 35154464 PMC8824885

[B50] Chen L. Zhan C.-Z. Wang T. You H. Yao R. (2020). Curcumin Inhibits the Proliferation, Migration, Invasion, and Apoptosis of Diffuse Large B-Cell Lymphoma Cell Line by Regulating miR-21/VHL axis. Yonsei Med. J. 61 (1), 20–29. 10.3349/ymj.2020.61.1.20 31887796 PMC6938780

[B51] Chen T. Yang C. Xi Z. Chen F. Li H. (2020). Reduced Caudal Type Homeobox 2 (CDX2) Promoter Methylation Is Associated with Curcumin’s Suppressive Effects on Epithelial-Mesenchymal Transition in Colorectal Cancer Cells. Med. Sci. Monit. 26, e926443. 10.12659/MSM.926443 32893845 PMC7496454

[B52] Chenxia H. Mengjie L. Tingting G. Shaoxi W. Weiping H. Ke Y. (2019). Anti-metastasis Activity of Curcumin against Breast Cancer via the Inhibition of Stem Cell-like Properties and EMT. Phytomedicine 58, 152740. 10.1016/j.phymed.2018.11.001 31005718

[B53] Cheriyamundath S. Ben-Ze’ev A. (2021). Wnt/β-catenin Target Genes in Colon Cancer Metastasis: the Special Case of L1CAM. Cancers 12, 3444. 10.3390/cancers12113444 PMC7699470 33228199

[B54] Chiu Y. -J. Tsai F. -J. Bau D. -T. Chang L. -C. Hsieh M. -T. Lu C. -C. (2021). Next-generation Sequencing Analysis Reveals that MTH-3, a Novel Curcuminoid Derivative, Suppresses the Invasion of MDA-MB-231 Triple-Negative Breast Adenocarcinoma Cells. Oncol. Rep. 46, 133. 10.3892/or.2021.8084 34013378 PMC8144931

[B55] Cianciosi D. Varela-Lopez A. Forbes-Hernandez T. Y. Gasparrini M. Afrin S. Reboredo-Rodriguez P. (2018). Targeting Molecular Pathways in Cancer Stem Cells by Natural Bioactive Compounds. Pharmacol. Res. 135, 150–165. 10.1016/j.phrs.2018.08.006 30103002

[B56] Cocker-Gurkan A. Celik M. Ugur M. Arisan E. D. Obakan-Yerlikaya P. Durdu Z. B. (2018). Curcumin Inhibits Autocrine Growth Hormone-Mediated Invasion and Metastasis by Targeting NF-κB Signaling and Polyamine Metabolism in Breast Cancer Cells. Amino Acids 50, 1045–1069. 10.1007/s00726-018-2581-z 29770869

[B57] Cui Y. Li L. Li Z. Yin J. Lane J. Ji J. (2021). Dual Effects of Targeting S100A11 on Suppressing Cellular Metastatic Properties and Sensitizing Drug Response in Gastric Cancer. Cancer Cell Int. 21, 243. 10.1186/s12935-021-01949-1 33931048 PMC8086328

[B58] Currie P. D. (1998). Hedgehog’s Escape from Pandora Box. J. Mol. Med. 76, 421–433. 10.1007/s001090050234 9625299

[B59] Dash S. Sahu A. K. Srivastava A. Chowdhury R. Mukherjee S. (2021). Exploring the Extensive Crosstalk between the Antagonistic Cytokines- TGF-β and TNF-α in Regulating Cancer Pathogenesis. Cytokine 138, 155348. 10.1016/j.cyto.2020.155348 33153895

[B60] De Francesco E. M. Maggiolini M. Musti A. M. (2018). Crosstalk between Notch, HIF-1α and GPER in Breast Cancer EMT. Int. J. Mol. Sci. 19, 2011. 10.3390/ijms19072011 PMC6073901 29996493

[B61] Debnath P. Huirem R. S. Dutta P. Palchaudhuri S. (2022). Epithelial-mesenchymal Transition and its Transcription Factors. Biosci. Rep. 42 (1), BSR20211754. 10.1042/BSR20211754 34708244 PMC8703024

[B62] Dev S. S. Abidin S. A. Z. Farghadani R. Othman I. Naidu R. (2021). Receptor Tyrosine Kinases and Their Signaling Pathways as Therapeutic Targets of Curcumin in Cancer. Front. Pharmacol. 12, 772510. 10.3389/fphar.2021.772510 34867402 PMC8634471

[B63] Dong B. Wu Y. (2021). Epigenetic Regulation and Post-translational Modifications of SNAI1 in Cancer Metastasis. Int. J. Mol. Sci. 22, 11062. 10.3390/ijms222011062 34681726 PMC8538584

[B64] Dong Y. Zheng Q. Wang Z. Lin X. You Y. Wu S. (2019). Higher Stiffness as an Independent Initiator Triggers Epithelial-Mesenchymal Transition and Facilitates HCC Metastasis. J. Hematol. Oncol. 12, 112. 10.1186/s13045-019-0795-5 31703598 PMC6839087

[B65] Dong Z. Feng Q. Zhang H. Liu Q. Gong J. (2021). Curcumin Enhances Drug Sensitivity of Gemcitabine-Resistant Lung Cancer Cells and Inhibits Metastasis. Pharmazie 76, 538–543. 10.1691/ph.2021.0927 34782038

[B66] Du T. Wang D. Wan X. Xu J. Xiao Q. Liu B. (2021). Regulatory Effect of microRNA-223-3p on Breast Cancer Cell Processes via the Hippo/Yap Signaling Pathway. Oncol. Lett. 22, 516. 10.3892/ol.2021.12777 33986876 PMC8114478

[B67] Duan Y. Chen H. -L. Ling M. Zhang S. Ma F. -X. Zhang H. -C. (2022). The Curcumin Analog EF24 Inhibits Proliferation and Invasion of Triple-Negative Breast Cancer Cells by Targeting the Long Noncoding RNA HCG11/Sp1 axis. Mol. Cell. Biol. 42 (1), e00163–21. 10.1128/mcb.00163-21 PMC8773073 34780286

[B68] Dudas A. Ladanyi A. Ingruber J. Steinbichler T. B. Riechelmann H. (2020). Epithelial to Mesenchymal Transition: a Mechanism that Fuels Cancer Radio/chemoresistance. Cells 9, 428. 10.3390/cells9020428 PMC7072371 32059478

[B69] Es H. A. Cox T. R. Sarafraz-Yazdi E. Thiery J. P. Warkiani M. E. (2021). Pirfenidone Reduces Epithelial-Mesenchymal Transition and Spheroid Formation in Breast Carcinoma through Targeting Cancer-Associated Fibroblasts (CAFs). Cancers 13, 5118. 10.3390/cancers13205118 34680267 PMC8533995

[B70] Faiella A. Riccardi F. Carteni G. Chiurazzi M. Onofrio L. (2022). The Emerging Role of C-Met in Carcinogenesis and Clinical Implications as a Possible Therapeutic Target. J. Oncol. 2022, 5179182. 10.1155/2022/5179182 35069735 PMC8776431

[B71] Fang F. Flegler A. J. Du P. Lin S. Clevenger C. V. (2009). Expression of Cyclophilin B Is Associated with Malignant Progression and Regulation of Genes Implicated in the Pathogenesis of Breast Cancer. Am. J. Pathol. 174 (1), 297–308. 10.2353/ajpath.2009.080753 19056847 PMC2631342

[B72] Fassina A. Cappellesso R. Guzzardo V. Dalla Via L. Piccolo S. Ventura L. (2012). Epithelial-mesenchymal Transition in Malignant Mesothelioma. Mod. Pathol. 25, 86–99. 10.1038/modpathol.2011.144 21983934

[B73] Feng Y. Guo X. Tang H. (2021). SLC6A8 Is Involved in the Progression of Non-small Cell Lung Cancer through the Notch Signaling Pathway. Ann. Transl. Med. 9 (3), 264. 10.21037/atm-20-5984 33708891 PMC7940877

[B74] Franci G. Miceli M. Altucci L. (2010). Targeting Epigenetic Networks with Polypharmacology: a New Avenue to Tackle Cancer. Epigenomics 2, 731–742. 10.2217/epi.10.62 22122079

[B75] Gaggioli C. (2008). Collective Invasion of Carcinoma Cells: when the Fibroblasts Take the Lead. Cell adh. Migr. 2 (1), 45–47. 10.4161/cam.2.1.5705 19262123 PMC2635002

[B76] Gall Trošelj K. Samaržija I. Tomljanović M. Kujundžić R. N. Đaković N. Mojzeš A. (2020). Implementing Curcumin in Translational Oncology Research. Molecules 25, 5240. 10.3390/molecules25225240 PMC7698148 33182817

[B77] Gallardo M. Calaf G. M. (2016). Curcumin Inhibits Invasive Capabilities through Epithelial Mesenchymal Transition in Breast Cancer Cell Lines. Int. J. Oncol. 49, 1019–1027. 10.3892/ijo.2016.3598 27573203

[B78] Gan G. N. Jimeno A. (2016). Emerging from Their Burrow: Hedgehog Pathway Inhibitors for Cancer. Exp. Opin. Investig. Drugs 25 (10), 1153–1166. 10.1080/13543784.2016.1216973 27459882

[B79] Gao C. Guo X. Xue A. Ruan Y. Wang H. Gao X. (2020). High Intratumoral Expression of eIF4A1 Promotes Epithelial-To-Mesenchymal Transition and Predicts Unfavorable Prognosis in Gastric Cancer. Acta Biochim. Biophys. Sin. 52 (3), 310–319. 10.1093/abbs/gmz168 32147684

[B80] Goustin A. S. Leof E. B. Shipley G. D. Moses H. L. (1986). Growth Factors and Cancer. Cancer Res. 46, 1015–1029. PMID: 3002607. 3002607

[B81] Gowhari Shabgah A. Hejri Zarifi S. Mazloumi Kiapey S. S. Ezzatifar F. Pahlavani N. Soleimani D. (2021). Curcumin and Cancer; Are Long Non-coding RNAs Missing Link? Prog. Biophys. Mol. Biol. 164, 63–71. 10.1016/j.pbiomolbio.2021.04.001 33894206

[B82] Grego-Bessa J. Diez J. de la Pompa J. L. (2004). Notch and Epithelial-Mesenchyme Transition in Development and Tumor Progression. Cell Cycle 3 (6), 718–721. 10.4161/cc.3.6.949 15197341

[B83] Gregory P. A. Bracken C. P. Bert A. G. Goodall G. J. (2008). MicroRNAs as Regulators of Epithelial-Mesenchymal Transition. Cell Cycle 7 (20), 3112–3118. 10.4161/cc.7.20.6851 18927505

[B84] Guo K. Wang P. Zhang L. Zhou Y. Dai X. Yan Y. (2021). Transcriptional Factor POU4F2 Promotes Colorectal Cancer Cell Migration and Invasion through Hedgehog-Mediated Epithelial-Mesenchymal Transition. Cancer Sci. 112, 4176–4186. 10.1111/cas.15089 34327778 PMC8486210

[B85] Habid J. G. O’Shaughnessy J. A. (2016). The Hedgehog Pathway in Triple-Negative Breast Cancer. Cancer Med. 5 (10), 2989–3006. 10.1002/cam4.833 27539549 PMC5083752

[B86] Han Y. Guo W. Ren T. Huang Y. Wang S. Liu K. (2019). Tumor-associated Macrophages Promote Lung Metastasis and Induce Epithelial-Mesenchymal Transition in Osteosarcoma by Activating the COX-2/STAT3 axis. Cancer Lett. 440-441, 116–125. 10.1016/j.canlet.2018.10.011 30343113

[B87] Han L. Wang S. Wei C. Fang Y. Huang S. Yin T. (2021). Tumour Microenvironment: a Non-negligible Driver for Epithelial-Mesenchymal Transition in Colorectal Cancer. Exp. Rev. Mol. Med. 23, e16. 10.1017/erm.2021.13 34758892

[B88] Hao Q. Wang P. Dutta P. Chung S. Li Q. Wang K. (2020). Comp34 Displays Potent Preclinical Antitumor Efficacy in Triple-Negative Breast Cancer via Inihibition of NUDT3-AS4, a Novel Oncogenic Long Noncoding RNA. Cell Death Dis. 11 (12), 1052. 10.1038/s41419-020-03235-w 33311440 PMC7733521

[B89] Hao J. Dai X. Gao J. Li Y. Hou Z. Chang Z. (2021). Curcumin Suppresses Colorectal Tumorigenesis via the Wnt/β-Catenin Signaling Pathway by Downregulating Axin2. Oncol. Lett. 21, 186. 10.3892/ol.2021.12447 33574925 PMC7816292

[B90] Hay E. D. (1995). An Overview of Epithelia-Mesenchymal Transformation. Acta Anat. (Basel) 154 (1), 8–20. 10.1159/000147748 8714286

[B91] He G. Feng C. Vinothkumar R. Chen W. Dai X. Chen X. (2016). Curcumin Analog EF24 Induces Apoptosis via ROS-dependent Mitochondrial Dysfunction in Human Colorectal Cancer Cells. Cancer Chemother. Pharmacol. 78, 1151–1161. 10.1007/s00280-016-3172-x 27787644

[B92] He G. Tianlong M. Yanshu P. Zhihua C. Qing X. Wenyan Y. (2018). Inhibitory Effect of DAPT on Notch Signaling Pathway in Curcumin Mediated Photodynamic Therapy for Cervical Cancer Xenografts in Nude Mice. Natl. Med. J. China 98 (19), 1511–1516. 10.3760/cma.j.issn.0376-2491.2018.19.012 29804421

[B93] He G. Tianlong M. Yuan Y. Yang W. Zhang Y. Chen Q. (2019). Effects of Notch Signaling Pathway in Cervical Cancer by Curcumin Mediated Photodynamic Therapy and its Possible Mechanisms *In Vitro* and *In Vivo* . J. Cancer 10, 4114–4122. 10.7150/jca.30690 31417656 PMC6692604

[B94] He H. Qiao K. Wang C. Yang W. Xu Z. Zhang Z. (2021). Hydrazinocurcumin Induces Apoptosis of Hepatocellular Carcinoma Cells through the P38 MAPK Pathway. Clin. Transl. Sci. 14, 2075–2084. 10.1111/cts.12765 32100959 PMC8504816

[B95] Höffken V. Hermann A. Pavenstädt H. Kremerskothen J. (2021). WWC Proteins: Important Regulators of Hippo Signaling in Cancer. Cancers 13, 306. 10.3390/cancers13020306 33467643 PMC7829927

[B96] Hu M. Du Q. Vancurova I. Lin X. Miller E. J. Simms H. H. (2005). Proapoptotic Effect of Curcumin on Human Neutrophils: Activation of the P38 Mitogen-Activated Protein Kinase Pathway. Crit. Care Med. 33 (11), 2571–2578. 10.1097/01.ccm.0000186760.20502.c7 16276182

[B97] Huang M. Fu M. Wang J. Xia C. Zhang H. Xiong Y. (2021). TGF-β1-activated Cancer-Associated Fibroblasts Promote Breast Cancer Invasion, Metastasis and Epithelial-Mesenchymal Transition by Autophagy or Overexpression of FAP-α. Biochem. Pharmacol. 188, 114527. 10.1016/j.bcp.2021.114527 33741330

[B98] Huang S. Lin W. Wang L. Gao Y. Yuan X. Zhang P. (2022). SIX1 Predicts Poor Prognosis and Facilitates the Progression of Non-small Lung Cancer via Activating the Notch Signaling Pathway. J. Cancer 13, 527–540. 10.7150/jca.61385 35069900 PMC8771509

[B99] Huber M. A. Kraut N. Beug H. (2005). Molecular Requirements for Epithelial-Mesenchymal Transition during Tumor Progression. Curr. Opin. Cell Biol. 17, 548–558. 10.1016/j.ceb.2005.08.001 16098727

[B100] Huminiecki L. Horbańczuk J. Atanasov A. G. (2017). The Functional Genomic of Curcumin. Semin. Cancer Biol. 46, 107–118. 10.1016/j.semcancer.2017.04.002 28392463

[B101] Hussen B. M. Shoorei H. Mohaqiq M. Dinger M. E. Hidayat H. J. Taheri M. (2021). The Impact of Non-coding RNAs in the Epithelial to Mesenchymal Transition. Front. Mol. Biosci. 8, 665199. 10.3389/fmolb.2021.665199 33842553 PMC8033041

[B102] Jahng Y. Park J. G. (2018). Recent Studies on Cyclic 1,7-diarylheptanoids: Their Isolation, Structures, Biological Activities, and Chemical Synthesis. Molecules 23, 3107. 10.3390/molecules23123107 PMC6321387 30486479

[B103] Jensen L. D. (2015). The Circadian Clock and Hypoxia in Tumor Cell De-differentiation and Metastasis. Biochim. Biophys. Acta 1850, 1633–1641. 10.1016/j.bbagen.2014.10.025 25450175

[B104] Jiang L. Zhang J. Xu Q. Wang B. Yao Y. Sun L. (2021). YAP Promotes the Proliferation and Migration of Colorectal Cancer Cells through the Glut3/AMPK Signaling Pathway. Oncol. Lett. 21, 312. 10.3892/ol.2021.12573 33692844 PMC7933749

[B105] Jiang M. Fang S. Zhao X. Zhou C. Gong Z. (2021). Epithelial-mesenchymal Transition-Related Circular RNAs in Lung Carcinoma. Cancer Biol. Med. 18, 411–420. 10.20892/j.issn.2095-3941.2020.0238 PMC8185863 33710806

[B106] Jiao D. Wang J. Lu W. Tang X. Chen J. Mou H. (2016). Curcumin Inhibited HGF-Induced EMT and Angiogenesis through Regulating C-Met Dependent PI3K/Akt/mTOR Signaling Pathways in Lung Cancer. Mol. Ther. Oncolytics 3, 16018. 10.1038/mto.2016.18 27525306 PMC4972091

[B107] Jin W. (2020). Role of JAK/STAT3 Signaling in the Regulation of Metastasis, the Transition of Cancer Stem Cells, and Chemoresistance of Cancer by Epithelial-Mesenchymal Transition. Cells 9, 217. 10.3390/cells9010217 PMC7017057 31952344

[B108] Jonckheere S. Adams J. De Groote D. Campbell K. Berx G. Goossens S. (2022). Epithelial-mesenchymal Transition (EMT) as a Therapeutic Target. Cells Tissue Organs 211, 157–182. 10.1159/000512218 33401271

[B109] Jones C. A. Hazlehurst L. A. (2021). Role of Calcium Homeostasis in Modulating EMT in Cancer. Biomedicines 9 (9), 1200. 10.3390/biomedicines9091200 34572386 PMC8471317

[B110] Kalafut J. Czerwonka A. Anameric A. Przybyszewska-Podstawska A. Misiorek J. O. Rivero-Müller A. (2021). Shooting at Moving and Hidden Targets – Tumour Cell Plasticity and the Notch Signalling Pathway in Head and Neck Squamous Cell Carcinomas. Cancers 13, 6219. 10.3390/cancers13246219 34944837 PMC8699303

[B111] Kallifatidis G. Hoy J. Lokeshwar B. L. (2016). Bioactive Natural Products for Chemoprevention and Treatment of Castration-Resistant Prostate Cancer. Semin. Cancer Biol. 40-41, 160–169. 10.1016/j.semcancer.2016.06.003 27370570 PMC5067195

[B338] Kaplan Z. Zielske S. P. Ibrahim K. G. Cackowski F. C. (2021). Wnt and b-catenin signaling in the bone metastasis of prostate cancer. Life 11, 1099. 10.3390/life11101099 34685470 PMC8537160

[B112] Kamp D. W. (2009). Asbestos-induced Lung Diseases: an Update. Transl. Res. 153 (4), 143–152. 10.1016/j.trsl.2009.01.004 19304273 PMC3544481

[B113] Kang Y. Massagué J. (2004). Epithelial-mesenchymal Transitions: Twist in Development and Metastasis. Cell 118, 277–279. 10.1016/j.cell.2004.07.011 15294153

[B114] Kang S. W. Rhee S. G. Chang T. -S. Jeong W. Choi M. H. (2005). 2-Cys Peroxiredoxin Function in Intracellular Signal Transduction: Therapeutic Implications. Trends Mol. Med. 11 (12), 571–578. 10.1016/j.molmed.2005.10.006 16290020 PMC7185838

[B115] Kar R. Jha N. K. Jha S. K. Sharma A. Dholpuria S. Asthana N. (2019). A « Notch » Deeper into the Epithelial-To-Mesenchymal Transition (EMT) Program in Breast Cancer. Genes 10, 961. 10.3390/genes10120961 PMC6947643 31766724

[B116] Karaosmanoğlu O. Banerjee S. Sivas H. (2018). Identification of Biomarkers Associated with Partial Epithelial to Mesenchymal Transition in the Secretome of Slug Over-expressing Hepatocellular Carcinoma Cells. Cell. Oncol. 41, 439–453. 10.1007/s13402-018-0384-6 PMC12995249 29858962

[B117] Karhadkar S. S. Bova G. S. Abdallah N. Dhara S. Gardner D. Maitra A. (2004). Hedgehog Signalling in Prostate Regeneration, Neoplasia, and Metastasis. Nature 431 (7009), 707–712. 10.1038/nature02962 15361885

[B118] Kaszak I. Witkowska-Pilaszewicz O. Niewiadomska Z. Dworecka-Kaszak B. Toka F. N. Jurka P. (2020). Role of Cadherins in Cancer – a Review. Int. J. Mol. Sci. 21, 7624. 10.3390/ijms21207624 PMC7589192 33076339

[B119] Kaur H. Moreau R. (2021). Curcumin Represses mTORC1 Signaling in Caco-2 Cells by a Two-Sided Mechanism Involving the Loss of IRS-1 and Activation of AMPK. Cell. Signal. 78, 109842. 10.1016/j.cellsig.2020.109842 33234350

[B120] Ke D. Y. J. El-Sahli S. Wang L. (2021). The Potential of Natural Products in the Treatment of Triple-Negative Breast Cancer. Curr. Cancer Drug Targets. 10.2174/1568009622666211231140623 34970954

[B121] Khan S. Setua S. Kumari S. Dan N. Massey A. Hafeez B. B. (2019). Superparamagnetic Iron Oxide Nanoparticles of Curcumin Enhance Gemcitabine Therapeutic Response in Pancreatic Cancer. Biomaterials 208, 83–97. 10.1016/j.biomaterials.2019.04.005 30999154 PMC13521448

[B122] Khan H. Ni Z. Feng H. Xing Y. Wu X. Huang D. (2021). Combination of Curcumin with N-N-Butyl Haloperidol Iodide Inhibits Hepatocellular Carcinoma Malignant Proliferation by Downregulating Enhancer of Zeste Homolog 2 (EZH2) – lncRNA H19 to Silence Wnt/β-Catenin Signaling. Phytomedicine 91, 153706. 10.1016/j.phymed.2021.153706 34517264

[B123] Khoontawad J. Intuyod K. Rucksaken R. Hongsrichan N. Pairojkul C. Pinlaor P. (2018). Discovering Proteins for Chemoprevention and Chemotherapy by Curcumin in Liver Fluke Infection-Induced Bile Duct Cancer. PLoS One 13 (11), e0207405. 10.1371/journal.pone.0207405 30440021 PMC6237386

[B124] Kim H. -J. Park J. -W. Cho Y. -S. Cho C. -H. Kim J. -S. Shin H. -W. (2013). Pathogenic Role of HIF-1α in Prostate Hyperplasia in the Presence of Chronic Inflammation. Biochim. Biophys. Acta 1832, 183–194. 10.1016/j.bbadis.2012.09.002 22986049

[B125] Kim M. -C. Hwang S. -H. Kim N. -Y. Lee H. -S. Ji S. Yang Y. (2018). Hypoxia Promotes Acquisition of Aggressive Phenotypes in Human Malignant Mesothelioma. BMC Cancer 18, 819. 10.1186/s12885-018-4720-z 30111297 PMC6094475

[B126] Kinoshita M. Kobayashi S. Gotoh K. Kubo M. Hayashi K. Iwagami Y. (2020). Heterogeneity of Treg/Th17 According to Cancer Progression and Modification in Biliary Tract Cancers via Self-Producing Cytokines. Dig. Dis. Sci. 65 (10), 2937–2948. 10.1007/s10620-019-06011-9 31853779

[B127] Koprowski S. Sokolowski K. Kunnimalaiyaan S. Gamblin T. C. Kunnimalaiyaan M. (2015). Curcumin-mediated Regulation of Notch1/hairy and Enhancer of Split-1/surviving: Molecular Targeting in Cholangiocarcinoma. J. Surg. Res. 198, 434–440. 10.1016/j.jss.2015.03.029 25890434

[B128] Kotiyal S. Bhattacharya S. (2015). Epithelial Mesenchymal Transition and Vascular Mimicry in Breast Cancer Stem Cells. Crit. Rev. Eukaryot. Gene Expr. 25 (3), 269–280. 10.1615/critreveukaryotgeneexpr.2015014042 26558950

[B129] Kumari N. Reabroi S. North B. J. (2021). Unraveling the Molecular Nexus between GPCRs, ERS, and EMT. Mediat. Inflamm. 2021, 6655417. 10.1155/2021/6655417 PMC7943314 33746610

[B130] Kumari N. Shonibare Z. Monavarian M. Arend R. C. Lee N. Y. Inman G. J. (2021b). TGF-β Signaling Networks in Ovarian Cancer Progression and Plasticity. Clin. Exp. Metast. 38, 139–161. 10.1007/s10585-021-10077-z PMC7987693 33590419

[B131] Laali K. K. Zwarycz A. T. Bunge S. D. Borosky G. L. Nukaya M. Kennedy G. D. (2019). Deuterated Curcuminoids: Synthesis, Structures, Computational/docking and Comparative Cell Viability Assays against Colorectal Cancer. Chem. Med. Chem. 14, 1173–1184. 10.1002/cmdc.201900179 30995360

[B132] Lee A. Y. -L. Fan C. -C. Chen Y. -A. Cheng C. -W. Sung Y. -J. Hsu C. -P. (2015). Curcumin Inhibits Invasiveness and Epithelial-Mesenchymal Transition in Oral Squamous Cell Carcinoma through Reducing Matrix Metalloproteinase 2, 9 and Modulating P53-E-Cadherin Pathway. Integr. Cancer Ther. 14 (5), 484–490. 10.1177/1534735415588930 26036622

[B133] Leggett S. E. Hruska A. M. Guo M. Wong I. Y. (2021). The Epithelial-Mesenchymal Transition and the Cytoskeleton in Bioengineered Systems. Cell Commun. Signal. 19, 32. 10.1186/s12964-021-00713-2 33691719 PMC7945251

[B134] Lehman H. L. Kidacki M. Stairs D. B. (2020). Twist2 Is NF-κB-Responsive when P120-Catenin Is Inactivated and EGFR Is Overexpressed in Esophageal Keratinocytes. Sci. Rep. 10, 18829. 10.1038/s41598-020-75866-0 33139779 PMC7608670

[B135] Lei Y. Chen L. Zhang G. Shan A. Ye C. Liang B. (2020). MicroRNAs Target the Wnt/β-Catenin Signaling Pathway to Regulate Epithelial-Mesenchymal Transition in Cancer (Review). Oncol. Rep. 44, 1299–1313. 10.3892/or.2020.7703 32700744 PMC7448411

[B136] Li Y. Zhang T. (2014). Targeting Cancer Stem Cells by Curcumin and Clinical Applications. Cancer Lett. 346 (2), 197–205. 10.1016/j.canlet.2014.01.012 24463298

[B137] Li Y. Maitah M. Y. Ahmad A. Kong D. Bao B. Sarkar F. H. (2012). Targeting the Hedgehog Signaling Pathway for Cancer Therapy. Expert. Opin. Ther. Targets 16 (1), 49–66. 10.1517/14728222.2011.617367 22243133

[B138] Li H. Wu H. Zhang H. Li Y. Li S. Hou Q. (2017). Identification of Curcumin-Inhibited Extracellular Matrix Receptors in Non-small Cell Lung Cancer A549 Cells by RNA Sequencing. Tumour Biol. 39 (6), 1010428317705334. 10.1177/1010428317705334 28618934

[B139] Li W. Sun L. Lei J. Wu Z. Ma Q. Wang Z. (2020). Curcumin Inhibits Pancreatic Cancer Cell Invasion and EMT by Interfering with Tumor-Stromal Crosstalk under Hypoxic Conditions via the IL-6/ERK/NF-κB axis. Oncol. Rep. 44 (1), 382–392. 10.3892/or.2020.7600 32377752

[B140] Li H. -L. Li Q. -Y. Jin M. -J. Lu C. -F. Mu Z. -Y. Xu W. -Y. (2021). A Review: Hippo Signaling Pathway Promotes Tumor Invasion and Metastasis by Regulating Target Gene Expression. J. Cancer Res. Clin. Oncol. 147 (6), 1569–1585. 10.1007/s00432-021-03604-8 33864521 PMC11801896

[B141] Li M. Guo T. Lin J. Huang X. Ke Q. Wu Y. (2022). Curcumin Inhibits the Invasion and Metastasis of Triple Negative Breast Cancer via Hedgehog/Gli1 Signaling Pathway. J. Ethnopharmacol. 283, 114689. 10.1016/j.jep.2021.114689 34592340

[B142] Li W. Jiang Z. Xiao X. Wang Z. Wu Z. Ma Q. (2018). Curcumin Inhibits Superoxide Dismutase-Induced Epithelial-Mesenchymal Transition via the PI3K/Akt/NF-κB Pathway in Pancreatic Cancer Cells. Int. J. Oncol. 52, 1593–1602. 10.3892/ijo.2018.4295 29512729

[B143] Li X. Wang X. Xie C. Zhu J. Meng Y. Chen Y. (2018). Sonic Hedgehog and Wnt/β-Catenin Pathways Mediate Curcumin Inhibition of Breast Cancer Stem Cells. Anticancer Drugs 29 (3), 208–215. 10.1097/CAD.0000000000000584 29356693

[B144] Li Y. Zu L. Wu H. Zhang F. Fan Y. Pan H. (2022). MiR-192/NKRF axis Confers Lung Cancer Cell Chemoresistance to Cisplatin via the NF-κB Pathway. Thorac. Cancer 13 (3), 430–441. 10.1111/1759-7714.14278 34953057 PMC8807278

[B145] Li Z. Gao Y. Li L. Xie S. (2022). Curcumin Inhibits Papillary Thyroid Cancer Cell Proliferation by Regulating lncRNA LINC00691. Anal. Cell. Pathol. 2022, 5946670. 10.1155/2022/5946670 PMC8898135 35256924

[B146] Liang Z. Li J. Zhao L. Deng Y. (2021). miR-375 Affects the Hedgehog Signaling Pathway by Downregulating RAC1 to Inhibit Hepatic Stellate Cell Viability and Epithelial-Mesenchymal Transition. Mol. Med. Rep. 23, 182. 10.3892/mmr.2020.11821 33398380 PMC7809903

[B147] Lim H. Koh M. Jin H. Bae M. Lee S. -Y. Kim K. M. (2021). Cancer-associated Fibroblasts Induce an Aggressive Phenotypic Shift in Non-malignant Breast Epithelial Cells via Interleukin-8 and S100A8. J. Cell Physiol. 236, 7014–7032. 10.1002/jcp.30364 33748944

[B148] Lin Y. -T. Wu K. -J. (2020). Epigenetic Regulation of Epithelial-Mesenchymal Transition: Focusing on Hypoxia and TGF-β Signaling. J. Biomed. Sci. 27, 39. 10.1186/s12929-020-00632-3 32114978 PMC7050137

[B149] Lin C. -C. Yang W. -H. Lin Y. -T. Tang X. Chen P. -H. Ding C. -K. C. (2021). DDR2 Upregulation Confers Ferroptosis Susceptibility of Recurrent Breast Tumors through the Hippo Pathway. Oncogene 40 (11), 2018–2034. 10.1038/s41388-021-01676-x 33603168 PMC7988308

[B150] Lin S. Zhang X. Huang G. Cheng L. Lv J. Zheng D. (2021). Myeloid-derived Suppressor Cells Promote Lung Cancer Metastasis by CCL11 to Activate ERK and AKT Signaling and Induce Epithelial-Mesenchymal Transition in Tumor Cells. Oncogene 40, 1476–1489. 10.1038/s41388-020-01605-4 33452453

[B151] Lin P. P. (2020). Aneuploid Circulating Tumor-Derived Endothelial Cell (CTEC): a Novel Versatile Player in Tumor Neovascularization and Cancer Metastasis. Cells 9, 1539. 10.3390/cells9061539 PMC7349247 32599893

[B152] Liu H. Yin J. Wang H. Jiang G. Deng M. Zhang G. (2015). FOXO3a Modulates WNT/β-catenin Signaling and Suppresses Epithelial-To-Mesenchymal Transition in Prostate Cancer Cells. Cell. Signal. 27, 510–518. 10.1016/j.cellsig.2015.01.001 25578861

[B153] Liu S. Chen S. Zeng J. (2018). TGF-β Signaling: a Complex Role in Tumorigenesis (Review). Mol. Med. Rep. 17, 699–704. 10.3892/mmr.2017.7970 29115550

[B154] Liu L. Wu Y. Zhang C. Zhou C. Li Y. Zeng Y. (2020). Cancer-associated Adipocyte-Derived G-CSF Promotes Breast Cancer Malignancy via STAT3 Signaling. J. Mol. Cell Biol. 12 (9), 723–737. 10.1093/jmcb/mjaa016 32242230 PMC7749739

[B155] Liu J. -M. Li M. Luo W. Sun H. -B. (2021). Curcumin Attenuates Adriamycin-Resistance of Acute Myeloid Leukemia by Inhibiting the lncRNA HOTAIR/miR-20a-5p/WT1 axis. Lab. Invest. 101 (10), 1308–1317. 10.1038/s41374-021-00640-3 34282279

[B156] Liu L. Lim M. A. Jung S. -N. Oh C. Won H. -R. Jin Y. L. (2021). The Effect of Curcumin on Multi-Level Immune Checkpoint Blockade and T Cell Dysfunction in Head and Neck Cancer. Phytomedicine 92, 153758. 10.1016/j.phymed.2021.153758 34592487

[B157] Liu X. Yin Z. Xu L. Liu H. Jiang L. Liu S. (2021). Upregulation of LINC01426 Promotes the Progression and Stemness in Lung Adenocarcinoma by Enhancing the Level of SHH Protein to Activate the Hedgehog Pathway. Cell Death Dis. 12, 173. 10.1038/s41419-021-03435-y 33568633 PMC7875967

[B158] Lopez-Bergami P. Barbero G. (2020). The Emerging Role of Wnt5a in the Promotion of a Pro-inflammatory and Immunosuppressive Tumor Microenvironment. Cancer metast. Rev. 39 (3), 933–952. 10.1007/s10555-020-09878-7 32435939

[B159] Lu Y. Zhang R. Zhang X. Zhang B. Yao Q. (2020). Curcumin May Reverse 5-fluorouracil Resistance on Colonic Cancer Cells by Regulating TET1-NKD-Wnt Signal Pathway to Inhibit the EMT Process. Biomed. Pharmacother. 129, 110381. 10.1016/j.biopha.2020.110381 32887024

[B160] Lu K. -H. Wu H. -H. Lin R. -C. Lin Y. -C. Lu P. W. -A. Yang S. -F. (2021). Curcumin Analogue L48H37 Suppresses Human Osteosarcoma U2OS and MG-63 Cells’ Migration and Invasion in Culture by Inhibition of uPA via the JAK/STAT Signaling Pathway. Molecules 26 (1), 30. 10.3390/molecules26010030 PMC7795127 33374783

[B161] Lu Y. Ding Y. Wei J. He S. Liu X. Pan H. (2021). Anticancer Effects of Traditional Chinese Medicine on Epithelial-Mesenchymal Transition (EMT) in Breast Cancer: Cellular and Molecular Targets. Eur. J. Pharmacol. 907, 174275. 10.1016/j.ejphar.2021.174275 34214582

[B162] Luo X. Cao M. Gao F. He X. (2021). YTHDF1 Promotes Hepatocellular Carcinoma Progression via Activating PI3K/AKT/mTOR Signaling Pathway and Inducing Epithelial-Mesenchymal Transition. Exp. Hematol. Oncol. 10, 35. 10.1186/s40164-021-00227-0 34088349 PMC8176587

[B163] Lv N. Liu F. Cheng L. Liu F. Kuang J. (2021). The Expression of Transcription Factors Is Different in Papillary Thyroid Cancer Cells during TNF-α Induced EMT. J. Cancer 12 (9), 2777–2786. 10.7150/jca.53349 33854637 PMC8040707

[B342] Lyu X. Li H. Ma X. Li X. Gao Y. Ni D. (2015). High-level S100A6 promotes metastasis and predicts the outcome of T1-T2 stage in clear cell renal cell carcinoma. Cell Biochem. Biophys 71, 279–290. 10.1007/s12013-014-0196-x 25120023

[B164] Ma Z. Xin Z. Hu W. Jiang S. Yang Z. Yan X. (2018). Forkhead Box O Proteins: Crucial Regulators of Cancer EMT. Semin. Cancer Biol. 50, 21–31. 10.1016/j.semcancer.2018.02.004 29427645

[B165] Ma J. Zhou C. Chen X. (2021). miR-636 Inhibits EMT, Cell Proliferation and Cell Cycle of Ovarian Cancer by Directly Targeting Transcription Factor Gli2 Involved in Hedgehog Pathway. Cancer Cell Int. 21, 64. 10.1186/s12935-020-01725-7 33472614 PMC7819188

[B166] Malik M. Mendoza M. Payson M. Catherino W. H. (2009). Curcumin, a Nutritional Supplement with Antineoplastic Activity, Enhances Leiomyoma Cell Apoptosis and Decreases Fibronectin Expression. Fertil. Steril. 91 (5), 2177–2184. 10.1016/j.fertnstert.2008.03.045 18555241

[B167] McCabe E. M. Rasmussen T. P. (2021). lncRNA Involvement in Cancer Stem Cell Function and Epithelial-Mesenchymal Transitions. Semin. Cancer Biol. 75, 38–48. 10.1016/j.semcancer.2020.12.012 33346133

[B168] McCarty M. F. (2012). Metformin May Antagonize Lin28 And/or Lin28B Activity, Thereby Boosting Let-7 Levels and Antagonizing Cancer Progression. Med. Hypotheses 78 (2), 262–269. 10.1016/j.mehy.2011.10.041 22129484

[B169] Meitzler J. L. Konaté M. M. Doroshow J. H. (2019). Hydrogen Peroxide-Producing NADPH Oxidases and the Promotion of Migratory Phenotypes in Cancer. Arch. Biochem. Biophys. 675, 108076. 10.1016/j.abb.2019.108076 31415727

[B170] Merikhian P. Eisavand M. R. Farahmand L. (2021). Triple-negative Breast Cancer: Understanding Wnt Signaling in Drug Resistance. Cancer Cell Int. 21, 419. 10.1186/s12935-021-02107-3 34376211 PMC8353874

[B171] Meyer T. Sand M. Schmitz L. Stockfleth E. (2021). The Role of Circular RNAs in Keratinocyte Carcinomas. Cancers 13, 4240. 10.3390/cancers13164240 34439394 PMC8392367

[B172] Mikula-Pietrasik J. Uruski P. Tykarski A. Ksiazek K. (2018). The Peritoneal “Soil” for a Cancerous “Seed”: a Comprehensive Review of the Pathogenesis of Intraperitoneal Cancer Metastases. Cell. Mol. Life Sci. 75, 509–525. 10.1007/s00018-017-2663-1 28956065 PMC5765197

[B173] Milton A. V. Konrad D. B. (2022). Epithelial-mesenchymal Transition and H_2_O_2_ Signaling – a Driver of Disease Progression and a Vulnerability in Cancers. Biol. Chem. 403 (4), 377–390. 10.1515/hsz-2021-0341 35032422

[B174] Mishra S. Patel S. Halpani C. G. (2019). Recent Updates in Curcumin Pyrazole and Isoxazole Derivatives: Synthesis and Biological Application. Chem. Biodivers. 16, e1800366. 10.1002/cbdv.201800366 30460748

[B175] Misiorek J. O. Przybyszewska-Podstawska A. Kalafut J. Paziewska B. Rolle K. Rivero-Müller A. (2021). Context Matters: NOTCH Signatures and Pathway in Cancer Progression and Metastasis. Cells 10, 94. 10.3390/cells10010094 PMC7827494 33430387

[B176] Mitra T. Bhattacharya R. (2020). Phytochemicals Modulate Cancer Aggressiveness: a Review Depicting the Anticancer Efficacy of Dietary Polyphenols and Their Combinations. J. Cell. Physiol. 235, 7696–7708. 10.1002/jcp.29703 32324275

[B177] Müller J. M. Rupec R. A. Baeuerle P. A. (1997). Study of Gene Regulation by NF-κB and AP-1 in Response to Reactive Oxygen Intermediates. Methods 11 (3), 301–312. 9073573 10.1006/meth.1996.0424

[B178] Müller T. Bain G. Wang X. Papkoff J. (2002). Regulation of Epithelial Cell Migration and Tumor Formation by B-Catenin Signaling. Exp. Cell Res. 280, 119–133. 10.1006/excr.2002.5630 12372345

[B179] Mukherjee A. Hollern D. P. Williams O. G. Rayburn T. S. Byrd W. A. Yates C. (2018). A Review of FOXI3 Regulation of Development and Possible Roles in Cancer Progression and Metastasis. Front. Cell Dev. Biol. 6, 69. 10.3389/fcell.2018.00069 30018953 PMC6038025

[B180] Nader J. Abadie J. Deshayes S. Boissard A. Blandin S. Blanquart C. (2018). Characterization of Increasing Stages of Invasiveness Identifies Stromal/cancer Cell Crosstalk in Rat Models of Mesothelioma. Oncotarget 9 (23), 16311–16329. 10.18632/oncotarget.24632 29662647 PMC5893242

[B181] Nader J. Guillon J. Petit C. Boissard A. Franconi F. Blandin S. (2020). S100A4 Is a Biomarker of Tumorigenesis, EMT, Invasion, and Colonization of Host Organs in Experimental Malignant Mesothelioma. Cancers 12, 939. 10.3390/cancers12040939 PMC7226589 32290283

[B182] Nagai T. Ishikawa T. Minami Y. Nishita M. (2020). Tactics of Cancer Invasion: Solitary and Collective Invasion. J. Biochem. 167 (4), 347–355. 10.1093/jb/mvaa003 31926018

[B183] Naujokat C. McKee D. L. (2021). The “Big Five” Phytochemicals Targeting Cancer Stem Cells: Curcumin, EGCG, Sulforaphane, Resveratrol and Genistein. Curr. Med. Chem. 28 (22), 4321–4342. 10.2174/0929867327666200228110738 32107991

[B184] Niehrs C. (2012). The Complex World of WNT Receptor Signalling. Nat. Rev. Mol. Cell. Biol. 13, 767–779. 10.1038/nrm3470 23151663

[B185] Niu Y. Shao Z. Wang H. Yang J. Zhang F. Luo Y. (2016). LASP1-S100A11 axis Promotes Colorectal Cancer Aggressiveness by Modulating TGFβ/Smad Signaling. Sci. Rep. 6, 26112. 10.1038/srep26112 27181092 PMC4867635

[B186] Niu G. Hao J. Sheng S. Wen F. (2022). Role of T-Box Genes in Cancer, Epithelial-Mesenchymal Transition, and Cancer Stem Cells. J. Cell. Biochem. 123, 215–230. 10.1002/jcb.30188 34897787

[B187] Oettgen H. F. Carswell E. A. Kassel R. L. Fiore N. Williamson B. Hoffmann M. K. (1980). Endotoxin-induced Tumor Necrosis Factor. Recent Results Cancer Res. 75, 207–212. 10.1007/978-3-642-81491-4_32 7232833

[B188] Ohashi Y. Tsuchiya Y. Koizumi K. Sakurai H. Saiki I. (2003). Prevention of Intrahepatic Metastasis by Curcumin in an Orthotopic Implantation Model. Oncology 65, 250–258. 10.1159/000074478 14657599

[B189] Onishi Y. Sakamoto T. Zhengguang L. Yasui H. Hamada H. Kubo H. (2020). Curcumin Inhibits Epithelial-Mesenchymal Transition in Oral Cancer Cells via C-Met Blockade. Oncol. Lett. 19, 4177–4182. 10.3892/ol.2020.11523 32391111 PMC7204627

[B190] Overholtzer M. Zhang J. Smolen G. A. Muir B. Li W. Sgroi D. C. (2006). Transforming Properties of YAP, a Candidate Oncogene on the Chromosome 11q22 Amplicon. Proc. Natl. Acad. Sci. U. S. A. 103 (33), 12405–12410. 10.1073/pnas.0605579103 16894141 PMC1533802

[B191] Pan G. Liu Y. Shang L. Zhou F. Yang S. (2021). EMT-Associated microRNAs and Their Roles in Cancer Stemness and Drug Resistance. Cancer Commun. (Lond) 41 (3), 199–217. 10.1002/cac2.12138 33506604 PMC7968884

[B192] Paolillo M. Schinelli S. (2019). Extracellular Matrix Alterations in Metastatic Processes. Int. J. Mol. Sci. 20, 4947. 10.3390/ijms20194947 PMC6802000 31591367

[B340] Papadaki M. A. Aggouraki D. Vetsika E. K. Xenidis N. Kallergi G. Kotsakis A. (2021). Epithelial-to-mesenchymal transition heterogeneity of circulating tumor cells and their correlation with MDSCs and Tregs in HER2-negative metastatic breast cancer patients. Anticancer Res 41 (2), 661–670. 10.21873/anticanres.14817 33517270

[B193] Paramanantham A. Kim M. J. Jung E. J. Nagappan A. Yun J. W. Kim H. J. (2020). Pretreatment of Anthocyanin from the Fruits of *Vitis Coignetiae Pulliat* Acts as a Potent Inhibitor of TNF-α Effect by Inhibiting NF-κB-Regulated Genes in Human Breast Cancer Cells. Molecules 25, 2396. 10.3390/molecules25102396 PMC7287973 32455624

[B194] Paramanantham A. Jung E. -J. Kim H. -J. Jeong B. -K. Jung J. -M. Kim G. -S. (2021). Doxorubicin-resistant TNBC Cells Exhibit Rapid Growth with Cancer Stem Cell-like Properties and EMT Phenotype, Which Can Be Transferred to Parental Cells through Autocrine Signaling. Int. J. Mol. Sci. 22, 12438. 10.3390/ijms222212438 34830320 PMC8623267

[B195] Parfenyev S. Singh A. Fedorova O. Daks A. Kulshrestha R. Barlev N. A. (2021). Interplay between P53 and Non-coding RNAs in the Regulation of EMT in Breast Cancer. Cell Death Dis. 12, 17. 10.1038/s41419-020-03327-7 33414456 PMC7791039

[B196] Park J. -I. Lee M. -G. Cho K. Park B. -J. Chae K. -S. Byun D. -S. (2003). Transforming Growth Factor-B1 Activates Interleukin-6 Expression in Prostate Cancer Cells through the Synergistic Collaboration of the Smad2, P38-NF-κB, JNK, and Ras Signaling Pathways. Oncogene 22, 4314–4332. 10.1038/sj.onc.1206478 12853969

[B197] Park J. W. Kim Y. Lee S. -B. Oh C. W. Lee E. J. Ko J. Y. (2022). Autophagy Inhibits Cancer Stemness in Triple-Negative Breast Cancer via miR-181a-Mediated Regulation of ATG5 And/or ATG2B. Mol. Oncol. 16 (9), 1857–1875. 10.1002/1878-0261.13180 35029026 PMC9067148

[B198] Paskeh M. D. A. Mirzaei S. Ashrafizadeh M. Zarrabi A. Sethi G. (2021). Wnt/β-catenin Signaling as a Driver of Hepatocellular Carcinoma Progression: an Emphasis on Molecular Pathways. J. Hepatocell. Carcinoma 8, 1415–1444. 10.2147/JHC.S336858 34858888 PMC8630469

[B199] Paskeh M. D. A. Mirzaei S. Guolami M. H. Zarrabi A. Zabolian A. Hashemi M. (2021b). Cervical Cancer Progression Is Regulated by SOX Transcription Factors: Revealing Signaling Networks and Therapeutic Strategies. Biomed. Pharmacother. 144, 112335. 10.1016/j.biopha.2021.112335 34700233

[B200] Patel S. Alam A. Pant R. Chattopadhyay S. (2019). Wnt Signaling and its Significance within the Tumor Microenvironment: Novel Therapeutic Insights. Front. Immunol. 10, 2872. 10.3389/fimmu.2019.02872 31921137 PMC6927425

[B201] Patiño-Morales C. Soto-Reyes E. Arechaga-Ocampo E. Ortiz-Sánchez E. Antonio-Véjar V. Pedraza-Chaverri J. (2020). Curcumin Stabilizes P53 by Interaction with NAD(P)H:quinone Oxidoreductase 1 in Tumor-Derived Cell Lines. Redox Biol. 28, 101320. 10.1016/j.redox.2019.101320 31526948 PMC6807312

[B202] Peinado H. Olmeda D. Cano A. (2007). Snail, ZEB and bHLH Factors in Tumour Progression: an Alliance against the Epithelial Phenotype? Nat. Rev. Cancer 7, 415–428. 10.1038/nrc2131 17508028

[B203] Peng P. -H. Hsu K., -W. Lai C. Y. J. Wu K., -J. (2021). The Role of Hypoxia-Induced Long Noncoding RNAs (lncRNAs) in Tumorigenesis and Metastasis. Biomed. J. 44, 521–533. 10.1016/j.bj.2021.03.005 34654684 PMC8640553

[B204] Peng Y. Zhang X. Lin H. Deng S. Qin Y. He J. (2021). Dual Activation of Hedgehog and Wnt/β-Catenin Signaling Pathway Caused by Downregulation of SUFU Targeted by miRNA-150 in Human Gastric Cancer. Aging 13 (7), 10749–10769. 10.18632/aging.202895 33848981 PMC8064165

[B205] Pouliquen D. L. Nawrocki-Raby B. Nader J. Blandin S. Robard M. Birembaut P. (2017). Evaluation of Intracavitary Administration of Curcumin for the Treatment of Sarcomatoid Mesothelioma. Oncotarget 8 (34), 57552–57573. 10.18632/oncotarget.15744 28915695 PMC5593667

[B206] Pouliquen D. L. Boissard A. Henry C. Blandin S. Richomme P. Coqueret O. (2020). Curcumin Treatment Identifies Therapeutic Targets within Biomarkers of Liver Colonization by Highly Invasive Mesothelioma Cells – Potential Links with Sarcomas. Cancers 12, 3384. 10.3390/cancers12113384 PMC7696465 33207594

[B207] Pouliquen D. L. Boissard A. Coqueret O. Guette C. (2020b). Biomarkers of Tumor Invasiveness in Proteomics (Review). Int. J. Oncol. 57, 409–432. 10.3892/ijo.2020.5075 32468071 PMC7307599

[B208] Pouliquen D. L. Boissard A. Henry C. Blandin S. Coqueret O. Guette C. (2021). Lymphoid Organ Proteomes Identify Therapeutic Efficacy Biomarkers Following the Intracavitary Administration of Curcumin in a Highly Invasive Rat Model of Peritoneal Mesothelioma. Int. J. Mol. Sci. 22, 8566. 10.3390/ijms22168566 34445271 PMC8395293

[B209] Principe D. R. Timbers K. E. Atia L. G. Koch R. M. Rana A. (2021). TGFβ Signaling in the Pancreatic Tumor Microenvironment. Cancers 13, 5086. 10.3390/cancers13205086 34680235 PMC8533869

[B210] Prud’homme G. J. (2012). Cancer Stem Cells and Novel Targets for Antitumor Strategies. Curr. Pharm. Des. 18 (19), 2838–2849. 10.2174/138161212800626120 22390767

[B211] Pu J. Huang Y. Fang Q. Wang J. Li W. Xu Z. (2021). Hypoxia-induced Fascin-1 Upregulation Is Regulated by Akt/Rac1 axis and Enhances Malignant Properties of Liver Cancer Cells via Mediating Actin Cytoskeleton Rearrangement and Hippo/YAP Activation. Cell Death Discov. 7, 385. 10.1038/s41420-021-00778-5 34897283 PMC8665929

[B212] Pucci M. Malagolini N. Dall’Olio F. (2021). Glycobiology of the Epithelial to Mesenchymal Transition. Biomedicines 9 (7), 770. 10.3390/biomedicines9070770 34356834 PMC8301408

[B213] Pustovalova M. Alhaddad L. Blokhina T. Smetanina N. Chigasova A. Chuprov-Netochin R. (2021). The CD44high Subpopulation of Multifraction Irradiation-Surviving NSCLC Cells Exhibits Partial EMT-Program Activation and DNA Damage Response Depending on Their P53 Status. Int. J. Mol. Sci. 22, 2369. 10.3390/ijms22052369 33673439 PMC7956695

[B214] Radtke F. Raj K. (2003). The Role of Notch in Tumorigenesis: Oncogene or Tumour Suppressor? Nat. Rev. Cancer 3 (10), 756–767. 10.1038/nrc1186 14570040

[B215] Ramamoorthi G. Sivalingam N. (2014). Molecular Mechanism of TGF-B Signaling Pathway in Colon Carcinogenesis and Status of Curcumin as Chemopreventive Strategy. Tumor Biol. 35, 7295–7305. 10.1007/s13277-014-1840-1 24668546

[B216] Ramundo V. Giribaldi G. Aldieri E. (2021). Transforming Growth Factor-β and Oxidative Stress in Cancer: a Crosstalk in Driving Tumor Transformation. Cancers 13, 3093. 10.3390/cancers13123093 34205678 PMC8235010

[B217] Ramundo V. Zanirato G. Aldieri E. (2021b). The Epithelial-To-Mesenchymal Transition (EMT) in the Development and Metastasis of Malignant Pleural Mesothelioma. Int. J. Mol. Sci. 22, 12216. 10.3390/ijms222212216 34830097 PMC8621591

[B218] Ravindran F. Koroth J. Manjunath M. Narayan S. Choudhary B. (2021). Curcumin Derivative ST09 Modulates the miR-199a-5p/DDR1 axis and Regulates Proliferation and Migration in Ovarian Cancer Cells. Sci. Rep. 11 (1), 23025. 10.1038/s41598-021-02454-1 34837026 PMC8626492

[B219] Ray T. Ryusaki T. Ray P. S. (2021). Therapeutically Targeting Cancers that Overexpress FOXC1: a Transcriptional Driver of Cell Plasticity, Partial EMT, and Cancer Metastasis. Front. Oncol. 11, 721959. 10.3389/fonc.2021.721959 34540690 PMC8446626

[B220] Rick J. W. Chandra A. Dalle Ore C. Nguyen A. T. Yagnik G. Aghi M. K. (2019). Fibronectin in Malignancy: Cancer-specific Alterations, Pro-tumoral Effects, and Therapeutic Implications. Semin. Oncol. 46 (3), 284–290. 10.1053/j.seminoncol.2019.08.002 31488338 PMC6801036

[B221] Rijsewijk F. Schuermann M. Wagenaar E. Parren P. Weigel D. Nusse R. (1987). The Drosophila Homolog of the Mouse Mammary Oncogene *Int-1* Is Identical to the Segment Polarity Gene Wingless. Cell 50 (4), 649–657. 10.1016/0092-8674(87)90038-9 3111720

[B222] Rokkam P. Gugalavath S. Kumar D. K. G. Vempati R. K. Malla R. R. (2020). Prognostic Rôle of Hedgehog-GLI1 Signaling Pathway in Aggressive and Metastatic Breast Cancers. Curr. Drug Metab. 21 (1), 33–43. 10.2174/1389200221666200122120625 31969097

[B223] Roy S. Sunkara R. R. Parmar M. Y. Shaikh S. Waghmare S. K. (2021). EMT Imparts Cancer Stemness and Plasticity: New Perspectives and Therapeutic Potential. Front. Biosci. (Landmark Ed.) 26, 238–265. 10.2741/4893 33049669

[B224] Ruiz de Porras V. Bystrup S. Martínez-Cardús A. Pluvinet R. Sumoy L. Howells L. (2016). Curcumin Mediates Oxaliplatin-Acquired Resistance Reversion in Colorectal Cancer Cell Lines through Modulation of CXC-chemokine/NF-κB Signalling Pathway. Sci. Rep. 6, 24675. 10.1038/srep24675 27091625 PMC4835769

[B225] Saho S. Satoh H. Kondo E. Inoue Y. Yamauchi A. Murata H. (2016). Active Secretion of Dimerized S100A11 Induced by the Peroxisome in Mesothelioma Cells. Cancer Microenviron. 9, 93–105. 10.1007/s12307-016-0185-2 27334300 PMC5264658

[B226] Sarkar F. H. Li Y. Wang Z. Kong D. (2010). The Role of Nutraceuticals in the Regulation of Wnt and Hedgehog Signaling in Cancer. Cancer Metastasis Rev. 29 (3), 383–394. 10.1007/s10555-010-9233-4 20711635 PMC2974632

[B227] Sato H. Sakaguchi M. Yamamoto H. Tomida S. Aoe K. Shien K. (2018). Therapeutic Potential of Targeting S100A11 in Malignant Pleural Mesothelioma. Oncogenesis 7, 11. 10.1038/s41389-017-0017-3 29362358 PMC5833371

[B228] Saunders J. A. Rogers L. A. C. Klomsiri C. Poole L. B. Daniel L. W. (2010). Reactive Oxygen Species Mediate Lysophosphatidic Acid Induced Signaling in Ovarian Cancer Cells. Free Radic. Biol. Med. 49 (12), 2058–2067. 10.1016/j.freeradbiomed.2010.10.663 20934509 PMC3005889

[B229] Savagner P. (2001). Leaving the Neighborhood: Molecular Mechanisms Involved during Epithelial-Mesenchymal Transition. Bioessays 23 (10), 912–923. 10.1002/bies.1132 11598958

[B230] Schelch K. Wagner C. Hager S. Pirker C. Siess K. Lang E. (2018). FGF2 and EGF Induce Epithelial-Mesenchymal Transition in Malignant Pleural Mesothelioma Cells via a MAPKinase/MMP1 Signal. Carcinogenesis 39 (4), 534–545. 10.1093/carcin/bgy018 29635378

[B231] Sementino E. Kadariya Y. Cheung M. Menges C. W. Tan Y. Kukuyan A. -M. (2022). Inactivation of P21-Activated Kinase 2 (Pak2) Inhibits the Development of *Nf2*-Deficient Tumors by Restricting Downstream Hedgehog and Wnt Signaling. Mol. Cell Res. 20 (5), 699–711. 10.1158/1541-7786.MCR-21-0837 PMC9081258 35082167

[B232] Sethi S. Li Y. Sarkar F. H. (2013). Regulating miRNA by Natural Agents as a New Strategy for Cancer Treatment. Curr. Drug Targets 14 (10), 1167–1174. 10.2174/13894501113149990189 23834152 PMC3899647

[B233] Shankar J. Messenberg A. Chan J. Underhill T. M. Foster L. J. Nabi I. R. (2010). Pseudopodial Actin Dynamics Control Epithelial-Mesenchymal Transition in Metastatic Cancer Cells. Cancer Res. 70 (9), 3780–3790. 10.1158/0008-5472.CAN-09-4439 20388789

[B234] Shao S. Duan W. Xu Q. Li X. Han L. Li W. (2019). Curcumin Suppresses Hepatic Stellate Cell-Induced Hepatocarcinoma Angiogenesis and Invasion through Downregulating CTGF. Oxid. Med. Cell. Long. 2019, 8148510. 10.1155/2019/8148510 PMC6360067 30800209

[B235] Shao Z. Gao D. Chen L. Ding W. Yu Q. (2020). Non-coding RNAs that Regulate the Wnt/β-Catenin Signaling Pathway in Gastric Cancer: Good Cop, Bad Cop? (Review). Oncol. Rep. 44 (4), 1314–1321. 10.3892/or.2020.7705 32945460

[B236] Shen H. Huang C. Wu J. Li J. Hu T. Wang Z. (2021). SCRIB Promotes Proliferation and Metastasis by Targeting Hippo/YAP Signalling in Colorectal Cancer. Front. Cell Dev. Biol. 9, 656359. 10.3389/fcell.2021.656359 33937255 PMC8084105

[B237] Shen Y., -W. Zhou Y., -D. Chen H., -Z. Luan X. Zhang W., -D. (2021). Targeting CTGF in Cancer: an Emerging Therapeutic Opportunity. Trends Cancer 7 (6), 511–524. 10.1016/j.trecan.2020.12.001 33358571

[B238] Shenoy A. K. Lu J. (2016). Cancer Cells Remodel Themselves and Vasculature to Overcome the Endothelial Barrier. Cancer Lett. 380, 534–544. 10.1016/j.canlet.2014.10.031 25449784 PMC4417104

[B239] Shi C. Chen Y. Chen Y. Yang Y. Bing W. Qi J. (2018). CD4+ CD25+ Regulatory T Cells Promote Hepatocellular Carcinoma Invasion via TGF-β1-Induced Epithelial-Mesenchymal Transition. OncoTargets Ther. 12, 279–289. 10.2147/OTT.S172417 PMC6314313 30643426

[B240] Shi J. Song S. Li S. Zhang K. Lan Y. Li Y. (2020). TNF-α/NF-κB Signaling Epigenetically Represses PSD4 Transcription to Promote Alcohol-Related Hepatocellular Carcinoma Progression. Cancer Med. 10, 3346–3357. 10.1002/cam4.3832 PMC8124102 33932127

[B241] Shimazu K. Inoue M. Sugiyama S. Fukuda K. Yoshida T. Taguchi D. (2018). Curcumin Analog, GO-Y078, Overcomes Resistance to Tumor Angiogenesis Inhibitors. Cancer Sci. 109, 3285–3293. 10.1111/cas.13741 30024080 PMC6172066

[B242] Shrestha R. Bridle K. R. Crawford D. H. G. Jayachandran A. (2020). TNF-α-mediated Epithelial-To-Msenchymal Transition Regulates Expression of Immune Checkpoint Molecules in Hepatocellular Carcinoma. Mol. Med. Rep. 21, 1849–1860. 10.3892/mmr.2020.10991 32319631 PMC7057769

[B243] Simeone P. Trerotola M. Franck J. Cardon T. Marchisio M. Fournier I. (2019). The Multiverse Nature of Epithelial to Mesenchymal Transition. Semin. Cancer Biol. 58, 1–10. 10.1016/j.semcancer.2018.11.004 30453041

[B244] Song X. Zhang M. Dai E. Luo Y. (2019). Molecular Targets of Curcumin in Breast Cancer (Review). Mol. Med. Rep. 19, 23–29. 10.3892/mmr.2018.9665 30483727

[B245] Stuelten C. H. Zhang Y. E. (2021). Transforming Growth Factor-β: an Agent of Change in the Tumor Microenvironment. Front. Cell Dev. Biol. 9, 764727. 10.3389/fcell.2021.764727 34712672 PMC8545984

[B246] Su C. -W. Chuang C. -Y. Chen Y. -T. Yang W. -E. Pan Y. -P. Lin C. -W. (2021). FLLL32 Triggers Caspase-Mediated Apoptotic Cell Death in Human Oral Cancer Cells by Regulating the P38 Pathway. Int. J. Mol. Sci. 22, 11860. 10.3390/ijms222111860 34769290 PMC8584525

[B247] Sun S. Fang H. (2021). Curcumin Inhibits Ovarian Cancer Progression by Regulating Circ-PLEKHM3/miR-320a/SMG1 axis. J. Ovarian Res. 14 (1), 158. 10.1186/s13048-021-00916-8 34784955 PMC8594156

[B248] Sun B. Zhong F. -J. (2021). ELTD1 Promotes Gastric Cancer Cell Proliferation, Invasion and Epithelial-Mesenchymal Transition through MAPK/ERK Signaling by Regulating CSK. Int. J. Gen. Med. 14, 4897–4911. 10.2147/IJGM.S325495 34475781 PMC8407680

[B249] Sun X., -D. Liu X., -E. Huang D., -S. (2013). Curcumin Reverses the Epithelial-Mesenchymal Transition of Pancreatic Cancer Cells by Inhibiting the Hedgehog Signaling Pathway. Oncol. Rep. 29, 2401–2407. 10.3892/or.2013.2385 23563640

[B250] Sun B. Zhang D. Zhao N. Zhao X. (2017). Epithelial-to-endothelial Transition and Cancer Stem Cells: Two Cornerstones of Vasculogenic Mimicry in Malignant Tumors. Oncotarget 8 (18), 30502–30510. 10.18632/oncotarget.8461 27034014 PMC5444760

[B251] Suresh R. Ali S. Ahmad A. Philip P. A. Sarkar F. H. (2016). The Role of Cancer Stem Cells in Recurrent and Drug-Resistant Lung Cancer. Adv. Exp. Med. Biol. 890, 57–74. 10.1007/978-3-319-24932-2_4 26703799

[B252] Syed I. S. Pedram A. Farhat W. A. (2016). Role of Sonic Hedgehog (Shh) Signaling in Bladder Cancer Stemness and Tumorigenesis. Curr. Urol. Rep. 17 (2), 11. 10.1007/s11934-015-0568-9 26757905

[B253] Takahashi K. Kobayashi M. Maishi N. Podyma-Inoue K. A. Hida K. Miyazono K. (2020). TNF-α Enhances TGF-β-Induced Endothelial-To-Mesenchymal Transition via TGF-β Signal Augmentation. Cancer Sci. 111, 2385–2399. 10.1111/cas.14455 32385953 PMC7385392

[B254] Takahashi K. Taniue K. Ono Y. Fujiya M. Mizukami Y. Okumura T. (2021). Long Non-coding RNAs in Epithelial-Mesenchymal Transition of Pancreatic Cancer. Front. Mol. Biosci. 8, 717890. 10.3389/fmolb.2021.717890 34820419 PMC8606592

[B255] Takehara M. Sato Y. Kimura T. Noda K. Miyamoto H. Fujino Y. (2020). Cancer-associated Adipocytes Promote Pancreatic Cancer Progression through SAA1 Expression. Cancer Sci. 111, 2883–2894. 10.1111/cas.14527 32535957 PMC7419047

[B256] Taki M. Abiko K. Ukita M. Murakami R. Yamanoi K. Yamaguchi K. (2021). Tumor Immune Microenvironment during Epithelial-Mesenchymal Transition. Clin. Cancer Res. 27, 4669–4679. 10.1158/1078-0432.CCR-20-4459 33827891

[B257] Takigawa H. Kitadai Y. Shinagawa K. Yuge R. Higashi Y. Tanaka S. (2017). Mesenchymal Stem Cells Induce Epithelial to Mesenchymal Transition in Colon Cancer Cells through Direct Cell-To-Cell Contact. Neoplasia 19 (5), 429–438. 10.1016/j.neo.2017.02.010 28433772 PMC5402629

[B258] Tang X. Sui X. Weng L. Liu Y. (2021). SNAIL1: Linking Tumor Metastasis to Immune Evasion. Front. Immunol. 12, 724200. 10.3389/fimmu.2021.724200 34917071 PMC8669501

[B259] Teeuwssen M. Fodde R. (2019). Wnt Signaling in Ovarian Cancer Stemness, EMT, and Therapy Resistance. J. Clin. Med. 8, 1658. 10.3390/jcm8101658 PMC6832489 31614568

[B260] Teicher B. A. Holden S. A. Ara G. Chen G. (1996). Transforming Growth Factor-β in *In Vivo* Resistance. Cancer Chemother. Pharmacol. 37, 601–609. 10.1007/s002800050435 8612316

[B261] Teicher B. A. Ikebe M. Ara G. Keyes S. R. Herbst R. S. (1997). Transforming Growth Factor-β1 Overexpression Produces Drug Resistance *In Vivo*: Reversal by Decorin. Vivo 11 (6), 463–472. PMID: 9509296. 9509296

[B262] Thompson-Elliott B. Johnson R. Khan S. A. (2021). Alterations in TGFβ Signaling during Prostate Cancer Progression. Am. J. Clin. Exp. Urol. 9 (4), 318–328. 34541030 PMC8446771

[B263] Thomson S. Petti F. Sujka-Kwok I. Mercado P. Bean J. Monaghan M. (2011). A Systems View of Epithelial-Mesenchymal Transition Signaling States. Clin. Exp. Metastasis 28, 137–155. 10.1007/s10585-010-9367-3 21194007 PMC3040305

[B264] Tian S. Liao L. Zhou Q. Huang X. Zheng P. Guo Y. (2021). Curcumin Inhibits the Growth of Liver Cancer by Impairing Myeloid-Derived Suppressor Cells in Murine Tumor Tissues. Oncol. Lett. 21, 286. 10.3892/ol.2021.12547 33732362 PMC7905673

[B265] Tiwari J. K. Negi S. Kashyap M. Nizamuddin S. Singh A. Khattri A. (2021). Pan-cancer Analysis Shows Enrichment of Macrophages, Overexpression of Checkpoint Molecules, Inhibitory Cytokines, and Immune Exhaustion Signatures in EMT-High Tumors. Front. Oncol. 11, 793881. 10.3389/fonc.2021.793881 35096592 PMC8790577

[B266] Toden S. Okugawa Y. Jascur T. Wodarz D. Komarova N. L. Buhrmann C. (2015). Curcumin Mediates Chemosensitization to 5-fluorouracil through miRNA-Induced Suppression of Epithelial-To-Mesenchymal Transition in Chemoresistant Colorectal Cancer. Carcinogenesis 36 (3), 355–367. 10.1093/carcin/bgv006 25653233 PMC4400529

[B267] Toledano M. B. Delaunay A. Monceau L. Tacnet F. (2004). Microbial H_2_O_2_ Sensors as Archetypical Redox Signaling Modules. Trends biochem. Sci. 29 (7), 351–357. 10.1016/j.tibs.2004.05.005 15236742

[B268] Topa J. Gresner P. Zaczek A. J. Markiewicz A. (2022). Breast Cancer Circulating Tumor Cells with Mesenchymal Features – an Unreachable Target? Cell. Mol. Life Sci. 79, 81. 10.1007/s00018-021-04064-6 35048186 PMC8770434

[B269] Tu S. P. Jin H. Shi J. D. Zhu L. M. Suo Y. Lu G. (2012). Curcumin Induces the Differentiation of Myeloid-Derived Suppressor Cells and Inhibits Their Interaction with Cancer Cells and Related Tumor Growth. Cancer Prev. Res. (Phila). 5 (2), 205–215. 10.1158/1940-6207.CAPR-11-0247 22030090 PMC3273601

[B270] Tu Y. Xie P. Du X. Fan L. Bao Z. Sun G. (2019). S100A11 Functions as Novel Oncogene in Glioblastoma via S100A11/ANXA/NF-κB Positive Feedback Loop. J. Cell Mol. Med. 23, 6907–6918. 10.1111/jcmm.14574 31430050 PMC6787445

[B271] Tuluhong D. Chen T. Wang J. Zeng H. Li H. Dunzhu W. (2021). FZD2 Promotes TGF-β-Induced Epithelial-To-Mesenchymal Transition in Breast Cancer via Activating Notch Signaling Pathway. Cancer Cell Int. 21, 199. 10.1186/s12935-021-01866-3 33832493 PMC8033683

[B272] Uthman Y. A. Ibrahim K. G. Abubakar B. Bello M. B. Malami I. Imam M. U. (2021). MALAT1: a Promising Therapeutic Target for the Treatment of Metastatic Colorectal Cancer. Biochem. Pharmacol. 190, 114657. 10.1016/j.bcp.2021.114657 34144008

[B273] Vallée A. Lecarpentier Y. Vallée J. -N. (2019). Curcumin: a Therapeutic Strategy in Cancers by Inhibiting the Canonical WNT/β-catenin Pathway. J. Exp. Clin. Cancer Res. 38, 323. 10.1186/s13046-019-1320-y 31331376 PMC6647277

[B274] van Meeteren L. A. ten Dijke P. (2012). Regulation of Endothelial Cell Plasticity by TGF-β. Cell Tissue Res. 347, 177–186. 10.1007/s00441-011-1222-6 21866313 PMC3250609

[B275] Varghese E. Samuel S. M. Abotaleb M. Cheema S. Mamtani R. Büsselberg D. (2018). The “Yin and Yang” of Natural Compounds in Anticancer Therapy of Triple-Negative Breast Cancers. Cancers 10 (10), 346. 10.3390/cancers10100346 PMC6209965 30248941

[B276] Venkatesh V. Nataraj R. Thangaraj G. S. Karthikeyan M. Gnanasekaran A. Kaginelli S. B. (2018). Targeting Notch Signalling Pathway of Cancer Stem Cells. Stem Cell Investig. 5, 5. 10.21037/sci.2018.02.02 PMC5897708 29682512

[B277] Vinod B. S. Maliekal T. T. Anto R. J. (2013). Phytochemicals as Chemosensitizers: from Molecular Mechanism to Clinical Significance. Antiox. Redox Signal. 18 (11), 1307–1348. 10.1089/ars.2012.4573 22871022

[B278] Vishnoi K. Vishwakarma N. Rana A. Rana B. (2020). Transcription Factors in Cancer Development and Therapy. Cancers 12, 2296. 10.3390/cancers12082296 PMC7464564 32824207

[B279] Wang C. Yang Z. Xu E. Shen X. Wang X. Li Z. (2021). Apolipoprotein C-II Induces EMT Topromote Gastric Cancer Peritoneal Metastasis via PI3K/AKT/mTOR Pathway. Clin. Transl. Med. 11, e522. 10.1002/ctm2.522 34459127 PMC8351524

[B280] Wang Z. Li Y. Ahmad A. Azmi A. S. Kong D. Banerjee S. (2010). Targeting miRNAs Involved in Cancer Stem Cells and EMT Regulation: an Emerging Concept in Overcoming Drug Resistance. Drug resist. Updat. 13 (4-5), 109–118. 10.1016/j.drup.2010.07.001 20692200 PMC2956795

[B281] Wang L. Wang C. Tao Z. Zhao L. Zhu Z. Wu W. (2019). Curcumin Derivative WZ35 Inhibits Tumor Cell Growth via ROS-YAP-JNK Signaling Pathway in Breast Cancer. J. Exp. Clin. Cancer Res. 38 (1), 460. 10.1186/s13046-019-1424-4 31703744 PMC6842168

[B282] Wang W. Xiao L. Pan D. Hu L. (2022). ASF1B Enhances Migration and Invasion of Lung Cancers Cell via Regulating the P53-Mediated Epithelial-Mesenchymal Transformation (EMT) Signaling Pathway. Neoplasma 69 (2), 361–369. 10.4149/neo_2021_210818N1181 35103478

[B283] Wang H. Zhang K. Liu J. Yang J. Tian Y. Yang C. (2021a). Curcumin Regulates Cancer Progression: Focus on ncRNAs and Molecular Signaling Pathways. Front. Oncol. 11, 660712. 10.3389/fonc.2021.660712 33912467 PMC8072122

[B284] Wang H. Zhang H. Sun Z. Chen W. Miao C. (2021b). GABAB Receptor Inhibits Tumor Progression and Epithelial-Mesenchymal Transition via the Regulation of Hippo/YAP1 Pathway in Colorectal Cancer. Int. J. Biol. Sci. 17, 1953–1962. 10.7150/ijbs.58135 34131398 PMC8193267

[B285] Wang H. Di X. Bi Y. Sun S. Wang T. (2021c). Long Non-coding RNA LINC00649 Regulates YES-Associated Protein 1 (YAP1)/Hippo Pathway to Accelerate Gastric Cancer (GC) Progression via Sequestering miR-16-5p. Bioengineered 12 (1), 1791–1802. 10.1080/21655979.2021.1924554 33975517 PMC8806528

[B286] Wang M. Liu X. Chen Z. Chen H. Tan Y. (2020). Metformin Suppressed Tumor Necrosis Factor-α-Induced Epithelial-Mesenchymal Transition in Prostate Cancer by Inactivating the NF-κB Signaling Pathway. Transl. Cancer Res. 9 (10), 6086–6095. 10.21037/tcr-20-1186 35117220 PMC8798386

[B287] Wang N. Feng T. Liu X. Liu Q. (2020). Curcumin Inhibits Migration and Invasion of Non-small Cell Lung Cancer Cells through Up-Regulation of miR-206 and Suppression of PI3K/AKT/mTOR Signaling Pathway. Acta Pharm. 70 (3), 399–409. 10.2478/acph-2020-0029 32074070

[B288] Wang P. Hao X. Li X. Yan Y. Tian W. Xiao L. (2021). Curcumin Inhibits Adverse Psychological Stress-Induced Proliferation and Invasion of Glioma Cells via Down-Regulating the ERK/MAPK Pathway. J. Cell. Mol. Med. 25, 7190–7203. 10.1111/jcmm.16749 34169637 PMC8335680

[B289] Wang W. Xie Y. Malhotra A. (2021). Potential of Curcumin and Quercetin in Modulation of Premature Mitochondrial Senescence and Related Changes during Lung Carcinogenesis. J. Environ. Pathol. Toxicol. Oncol. 40 (4), 53–60. 10.1615/JEnvironPatholToxicolOncol.2021039371 34936300

[B290] Wang Y. Lan W. Xu M. Song J. Mao J. Li C. (2021). Cancer-associated Fibroblast-Derived SDF-1 Induces Epithelial-Mesenchymal Transition in Lung Adenocarcinoma via CXCR4/β-catenin/PPARδ Signalling. Cell Death Dis. 12, 214. 10.1038/s41419-021-03509-x 33637678 PMC7910618

[B291] Wang Z. Liu F. Liao W. Yu L. Hu Z. Li M. (2020). Curcumin Suppresses Glioblastoma Cell Proliferation by P-AKT/mTOR Pathway and Increases the PTEN Expression. Arch. Biochem. Biophys. 689, 108412. 10.1016/j.abb.2020.108412 32445778

[B292] Wang Z. Ao X. Shen Z. Ao L. Wu X. Pu C. (2021a). TNF-α Augments CXCL10/CXCR3 axis Activity to Induce Epithelial-Mesenchymal Transition in Colon Cancer Cell. Int. J. Biol. Sci. 17, 2683–2702. 10.7150/ijbs.61350 34345201 PMC8326125

[B293] Wang Z. Wang Y. Ma X. Dang C. (2021b). RSPO2 Silence Inhibits Tumorigenesis of Nasopharyngeal Carcinoma by ZNRF3/Hedgehog-Gli1 Signal Pathway. Life Sci. 282, 119817. 10.1016/j.lfs.2021.119817 34273374

[B294] Wieczorek-Szukala K. Lewinski A. (2021). The Role of Snail-1 in Thyroid Cancer – what We Know So Far. J. Clin. Med. 10, 2324. 10.3390/jcm10112324 34073413 PMC8197874

[B295] Xia Y. Weng B. Wang Z. Kang Y. Shi L. Huang G. (2016). W346 Inhibits Cell Growth, Invasion, Induces Cycle Arrest and Potentiates Apoptosis in Human Gastric Cancer Cells *In Vitro* through the NF-κB Signaling Pathway. Tumor Biol. 37, 4791–4801. 10.1007/s13277-015-4277-2 26520440

[B296] Xiao L. Peng H. Yan M. Chen S. (2021). Silencing *ACTG1* Expression Induces Prostate Cancer Epithelial Mesenchymal Transition through *MAPK/ERK* Signaling Pathway. DNA Cell Biol. 40 (11), 1445–1455. 10.1089/dna.2021.0416 34767732

[B297] Xie C. Zhu J. Yang X. Huang C. Zhou L. Meng Z. (2021). TAp63a Is Involved in Tobacco Smoke-Induced Lung Cancer EMT and the Anti-cancer Activity of Curcumin via miR-19 Transcriptional Suppression. Front. Cell Dev. Biol. 9, 645402. 10.3389/fcell.2021.645402 33748140 PMC7970191

[B298] Xu X. Su B. Xie C. Wei S. Zhou Y. Liu H. (2014). Sonic Hedgehog-Gli1 Signaling Pathway Regulates the Epithelial Mesenchymal Transition (EMT) by Mediating a New Target Gene, S100A4, in Pancreatic Cancer Cells. PLoS One 9 (7), e96441. 10.1371/journal.pone.0096441 25072505 PMC4114558

[B299] Xu F. Ji Z. He L. Chen M. Chen H. Feng Q. (2020). Downregulation of LINCO1021 by Curcumin Analog Da0324 Inhibits Gastric Cancer Progression through Activation of P53. Am. J. Transl. Res. 12 (7), 3429–3444. 32774710 PMC7407729

[B300] Xu H. Dun S. Gao Y. Ming J. Hui L. Qiu X. (2021). TMEM107 Inhibits EMT and Invasion of NSCLC through Regulating the Hedgehog Pathway. Thorac. Cancer 12 (1), 79–89. 10.1111/1759-7714.13715 33124203 PMC7779196

[B301] Xu J. Fang X. Long L. Wang S. Qian S. Lyu J. (2021). HMGA2 Promotes Breast Cancer Metastasis by Modulating Hippo-YAP Signaling Pathway. Cancer Biol. Ther. 22 (1), 5–11. 10.1080/15384047.2020.1832429 33307962 PMC7834087

[B302] Xue L. Tao Y. Yuan Y. Qu W. Wang W. (2021). Curcumin Suppresses Renal Carcinoma Tumorigenesis by Regulating Circ-FNDC3B/miR-138-5p/IGF2 axis. Anticancer Drugs 32 (7), 734–744. 10.1097/CAD.0000000000001063 34001703

[B303] Yan G. Chang Z. Wang C. Gong Z. Xin H. Liu Z. (2022). LncRNA ILF3-AS1 Promotes Cell Migration, Invasion and EMT Process in Hepatocellular Carcinoma via the miR-628-5p/MEIS2 axis to Activate the Notch Pathway. Dig. Liver Dis. 54, 125–135. 10.1016/j.dld.2021.04.036 34053876

[B304] Yang J. Zhu D. Liu S. Shao M. Liu Y. Li A. (2020). Curcumin Enhances Radiosensitization of Nasopharyngeal Carcinoma by Regulating circRNA Network. Mol. Carcinog. 59 (2), 202–214. 10.1002/mc.23143 31793078

[B305] Yang M. Han Y. -m. Han Q. Rong X. -z. Liu X. -f. Ln X. -Y. (2021). KCTD11 Inhibits Progression of Lung Cancer by Binding to β-catenin to Regulate the Activity of the Wnt and Hippo Pathways. J. Cell Mol. Med. 25, 9411–9426. 10.1111/jcmm.16883 34453479 PMC8500973

[B306] Yao J. Lin J. He L. Huang J. Liu Q. (2020). TNF-α/miR-155 axis Induces the Transformation of Osteosarcoma Cancer Stem Cells Independent of TP53INP1. Gene 726, 144224. 10.1016/j.gene.2019.144224 31669646

[B307] Ye C. Wang W. Xia G. Yu C. Yi Y. Hua C. (2019). A Novel Curcumin Derivative CL-6 Exerts Antitumor Effect in Human Gastric Cancer Cells by Inducing Apoptosis through Hippo-YAP Signaling Pathway. OncoTargets Ther. 12, 2259–2269. 10.2147/OTT.S196914 PMC6441554 30988630

[B308] Yeap S. K. Mohd Ali N. Akhtar M. N. Razak N. A. Chong Z. X. Ho W. Y. (2021). Induction of Apoptosis and Regulation of microRNA Expression by (2E,6E)-2,6-Bis-(4-Hydroxy-3-Methoxybenzylidene)-Cyclohexanone (BHMC) Treatment on MCF-7 Breast Cancer Cells. Molecules 26 (5), 1277. 10.3390/molecules26051277 33652854 PMC7956517

[B309] Yoshimatsu Y. Watabe T. (2022). Emerging Roles of Inflammation-Mediated Endothelial-Mesenchymal Transition in Health and Disease. Inflamm. Regen. 42 (1), 9. 10.1186/s41232-021-00186-3 35130955 PMC8818500

[B310] You X. Wu J. Zhao X. Jiang X. Tao W. Chen Z. (2021). Fibroblastic Galectin-1-Fostered Invasion and Metastasis Are Mediated by TGF-β1-Induced Epithelial-Mesenchymal Transition in Gastric Cancer. Aging (Albany NY) 13 (14), 18464–18481. 10.18632/aging.203295 34260413 PMC8351703

[B311] Yu X. Yustein J. T. Xu J. (2021). Research Models and Mesenchymal/epithelial Plasticity of Osteosarcoma. Cell Biosci. 11, 94. 10.1186/s13578-021-00600-w 34022967 PMC8141200

[B312] Zavadil J. Narasimhan M. Blumenberg M. Schneider R. J. (2007). Transforming Growth Factor-β and microRNA:mRNA Regulatory Networks in Epithelial Plasticity. Cells Tissue Organs 185, 157–161. 10.1159/000101316 17587821

[B313] Zeng Y. Du Q. Zhang Z. Ma J. Han L. Wang Y. (2020). Curcumin Promotes Cancer-Associated Fibroblasts Apoptosis via ROS-Mediated Endoplasmic Reticulum Stress. Arch. Biochem. Biophys. 694, 108613. 10.1016/j.abb.2020.108613 33010228

[B314] Zhang Y. Weinberg R. A. (2018). Epithelial-to-mesenchymal Transition in Cancer: Complexity and Opportunities. Front. Med. 12 (4), 361–373. 10.1007/s11684-018-0656-6 30043221 PMC6186394

[B315] Zhang C. Berndt-Paetz M. Neuhaus J. (2021). A Comprehensive Bioinformatics Analysis of Notch Pathways in Bladder Cancer. Cancers 13, 3089. 10.3390/cancers13123089 34205690 PMC8235546

[B316] Zhang J. Ji J. -Y. Yu M. Overholtzer M. Smolen G. A. Wang R. (2009). YAP-Dependent Induction of Amphiregulin Identifies a Non-cell-autonomous Component of the Hippo Pathway. Nat. Cell Biol. 11 (12), 1444. 10.1038/ncb1993 19935651 PMC2819909

[B317] Zhang Q. Ye Z. Yang Q. He X. Wang H. Zhao Z. (2012). Upregulated Expression of Annexin II Is a Prognostic Marker for Patients with Gastric Cancer. World J. Surg. Oncol. 10, 103. 10.1186/1477-7819-10-103 22681645 PMC3433344

[B318] Zhang J. Zhang K. Jiang X. Zhang J. (2014). S100A6 as a Potential Serum Prognostic Biomarker and Therapeutic Target in Gastric Cancer. Dig. Dis. Sci. 59, 2136–2144. 10.1007/s10620-014-3137-z 24705642

[B319] Zhang L. Cheng X. Gao Y. Zhang C. Bao J. Guan H. (2016). Curcumin Inhibits Metastasis in Human Papillary Thyroid Carcinoma BCPAP Cells via Down-Regulation of the TGF-β/Smad2/3 Signaling Pathway. Exp. Cell Res. 341, 157–165. 10.1016/j.yexcr.2016.01.006 26826337

[B320] Zhang M. Zheng S. Jing C. Zhang J. Shen H. Xu X. (2018). S100A11 Promotes TGF-β1-Induced Epithelial-Mesenchymal Transition through SMAD2/3 Signaling Pathway in Intrahepatic Cholangiocarcinoma. Future Oncol. 14 (9), 837–847. 10.2217/fon-2017-0534 29569474

[B321] Zhang H. -H. Li R. Li Y. -J. Yu X. -X. Sun Q. -N. Li A. -Y. (2020). eIF4E-related miR-320a and miR-340-5p Inhibit Endometrial Carcinoma Cell Metastatic Capability by Preventing TGF-β1-Induced Epithelial-Mesenchymal Transition. Oncol. Rep. 43, 447–460. 10.3892/or.2019.7437 31894279 PMC6967095

[B322] Zhang J. -Y. Zhu W. -W. Wang M. -Y. Zhai R. -D. Wang Q. Shen W. -L. (2021). Cancer-associated Fibroblasts Promote Oral Squamous Cell Carcinoma Progression through LOX-Mediated Matrix Stiffness. J. Transl. Med. 19, 513. 10.1186/s12967-021-03181-x 34930321 PMC8686394

[B323] Zhang M. Gan W. Jing C. -Y. Zheng S. -S. Yi Y. Zhang J. (2019). S100A11 Promotes Cell Proliferation via P38/MAPK Signaling Pathway in Intrahepatic Cholangiocarcinoma. Mol. Carcinog. 58, 19–30. 10.1002/mc.22903 30182496 PMC6587853

[B324] Zhang N. Ji J. Zhou D. Liu X. Zhang X. Liu Y. (2021). The Interaction of the Senescent and Adjacent Breast Cancer Cells Promotes the Metastasis of Heterogeneous Breast Cancer Cells through Notch Signaling. Int. J. Mol. Sci. 22, 849. 10.3390/ijms22020849 PMC7830992 33467780

[B325] Zhang T. Zheng P. Shen X. Shao R. Wang B. Shen H. (2019). Curcuminoid WZ26, a TrxR1 Inhibitor, Effectively Inhibits Colon Cancer Cell Growth and Enhances Cisplatin-Induced Cell Death through the Induction of ROS. Free Radic. Biol. Med. 141, 93–102. 10.1016/j.freeradbiomed.2019.06.005 31176737

[B326] Zhang X. Zhang C. Ren Z. Zhang F. Xu J. Zhang X. (2020). Curcumin Affects Gastric Cancer Cell Migration, Invasion and Cytoskeletal Remodeling through Gli1-β-Catenin. Cancer Manag. Res. 12, 3795–3806. 10.2147/CMAR.S244384 32547215 PMC7247599

[B327] Zhang Y. -L. Ma Y. Zeng Y. -Q. Liu Y. He E. -P. Liu Y. -T. (2021). A Narrative Review of Research Progress on FoxM1 in Breast Cancer Carcinogenesis and Therapeutics. Ann. Transl. Med. 9 (22), 1704. 10.21037/atm-21-5271 34988213 PMC8667115

[B328] Zhao J. -a. Nie W. Dong L. Liu W. Wei W. (2021). A Curcumin Analog GL63 Inhibits the Malignant Behaviors of Hepatocellular Carcinoma by Inactivating the JAK2/STAT3 Signaling Pathway via the Circular RNA Zinc Finger Protein 83/microRNA-334-5p/cyclin-dependent Kinase 16 axis. J. Gastroenterol. Hepatol. 36 (10), 2967–2977. 10.1111/jgh.15545 PMC8518784 33982329

[B329] Zhao Y. Wang Y. Chen W. Bai S. Peng W. Zheng M. (2021). Targeted Intervention of eiF4A1 Inhibits EMT and Metastasis of Pancreatic Cancer Cells via C-MYC/miR-9 Signaling. Cancer Cell Int. 21, 670. 10.1186/s12935-021-02390-0 34906136 PMC8672469

[B330] Zheng Y. Yang X. Tan J. Tian R. Shen P. Cai W. (2020). Curcumin Suppresses the Stemness of Non-small Cell Lung Cancer Cells via Promoting the Nuclear-Cytoplasm Translocation of TAZ. Environ. Toxicol. 36, 1135–1142. 10.1002/tox.23112 33539684

[B331] Zheng H. Liu H. Li H. Dou W. Wang X. (2021). Weighed Gene Co-expression Network Analysis Identifies a Cancer-Associated Fibroblast Signature for Predicting Prognosis and Therapeutic Responses in Gastric Cancer. Front. Mol. Biosci. 8, 744677. 10.3389/fmolb.2021.744677 34692770 PMC8531434

[B332] Zheng N. Zhang S. Wu W. Zhang N. Wang J. (2021). Regulatory Mechanisms and Therapeutic Targeting of Vasculogenic Mimicry in Hepatocellular Carcinoma. Pharmacol. Res. 166, 105507. 10.1016/j.phrs.2021.105507 33610718

[B333] Zhou D. He L. (2021). Sauchinone Inhibits Hypoxia-Induced Invasion and Epithelial-Mesenchymal Transition in Osteosarcoma Cells via Inactivation of the Sonic Hedgehog Pathway. J. Recept. Signal Transduct. Res. 42, 173–179. 10.1080/10799893.2021.1881556 33563062

[B334] Zhou L. Li J. Tang Y. Yang M. (2021). Exosomal lncRNA LINC00659 Transferred from Cancer-Associated Fibroblasts Promotes Colorectal Cancer Cell Progression via miR-342-3p/ANXA2 axis. J. Transl. Med. 19, 8. 10.1186/s12967-020-02648-7 33407563 PMC7789760

[B335] Zhou G. -Z. Guo S. -S. Liu D. -X. Zhang L. Sun G. -C. (2020). Antiproliferative Effect and Autophagy Induction of Curcumin Derivative ZYX02-Na on the Human Lung Cancer Cells A549. J. Biochem. Mol. Toxicol. 34 (12), e22592. 10.1002/jbt.22592 33176062

[B336] Zhou L. Lu Y. Liu J. -S. Long S. -Z. Liu H. -L. Zhang J. (2020). The Rôle of miR-21/RECK in the Inhibition of Osteosarcoma by Curcumin. Mol. Cell. Probes 51, 101534. 10.1016/j.mcp.2020.101534 32081769

[B337] Zhou Q. Wu X. Wang X. Yu Z. Pan T. Li Z. (2020). The Reciprocal Interaction between Tumor Cells and Activated Fibroblasts Mediated by TNF-α/IL-33/ST2L Signaling Promotes Gastric Cancer Metastasis. Oncogene 39, 1414–1428. 10.1038/s41388-019-1078-x 31659258 PMC7018661

